# Saproxylic insects and fungi in deciduous forests along a rural–urban gradient

**DOI:** 10.1002/ece3.7152

**Published:** 2021-01-27

**Authors:** Sandro Meyer, Hans‐Peter Rusterholz, Bruno Baur

**Affiliations:** ^1^ Department of Environmental Sciences Section of Conservation Biology University of Basel Basel Switzerland

**Keywords:** beetles, fine woody debris, flies, forest size, urbanization

## Abstract

Urbanization is increasing worldwide and is regarded a major threat to biodiversity in forests. As consequences of intensive human use, the vegetation structure of naturally growing urban forests and their amount of deadwood can be reduced. Deadwood is an essential resource for various saproxylic insects and fungi. We assessed the effects of urbanization and forest characteristics on saproxylic insects and fungi. We exposed standardized bundles consisting of each three freshly cut beech and oak branches in 25 forests along a rural–urban gradient in Basel (Switzerland). After an exposure of 8 months, we extracted the saproxylic insects for 10 months using an emergence trap for each bundle. We used drilling chips from each branch to determine fungal operational taxonomic units (OTUs). In all, 193,534 insect individuals emerged from the experimental bundles. Our study showed that the abundance of total saproxylic insects, bark beetles, longhorn beetles, total flies, moths, and ichneumonid wasps decreased with increasing degree of urbanization, but not their species richness. However, the taxonomic composition of all insect groups combined was altered by wood moisture of branches and that of saproxylic beetles was influenced by the degree of urbanization. Unexpectedly, forest size and local forest characteristics had a minor effect on saproxylic insects. ITS (internal transcribed spacer of rDNA) analysis with fungal specific primers revealed a total of 97 fungal OTUs on the bundles. The number of total fungal OTUs decreased with increasing degree of urbanization and was affected by the volume of naturally occurring fine woody debris. The composition of fungal OTUs was altered by the degree of urbanization and pH of the branch wood. As a consequence of the altered compositions of saproxylics, the association between total saproxylic insects and fungi changed along the rural–urban gradient. Our study shows that urbanization can negatively impact saproxylic insects and fungi.

## Graphical abstract

We assessed the effects of urbanization, forest size and forest site characteristics on saproxylic insects and fungi using freshly cut beech and oak branches. We found that the degree of urbanization, forest site and branch characteristics can impact the number of individuals of several saproxylic insect groups and the number of fungal OTUs found on the branches. The degree of urbanization also altered the composition of saproxylic beetles and fungal OTUs.

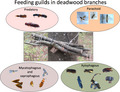

## 1 INTRODUCTION

Urbanization is increasing globally and is considered a major driver of environmental change (Grimm et al., 2008). The expansion of built‐up areas reduces the size of natural and seminatural areas and increases their spatial isolation (McKinney, 2006). Several studies reported alterations in abiotic conditions in remaining habitat patches (e.g., increased temperature and N deposition) from the rural surroundings to the city center (Pickett et al., 2011). These changes influence habitat quality and, consequently, the species richness, species composition, and functional diversity of plants and arthropods, which in turn affect the functioning of ecosystems (McDonnell & Hahs, 2015; Merckx & Van Dyck, 2019). Furthermore, urbanization can influence the population dynamics of animals and plants by altering the distribution and abundance of hosts, pathogens, and vectors (Meineke et al., 2013; Moreira et al., 2019).

Urban forests, which can be either remnants of former continuous forests, actively planted, or a result of ongoing succession, are among the most frequent type of green areas in cities (Cvejić et al., 2015). Urban forests provide a wide range of ecosystem services for residents, from areas for recreation, to the recycling and storage of nutrients, air filtering and temperature regulation, and habitat for native species (Escobedo et al., 2011). In general, temperate broad‐leaved forests in Europe have been more heavily impacted compared to any other forest biome (Hannah et al., 1995). Naturally growing urban forests can vary in intensity of recreational activities and maintenance measures (New, 2015). As a result of intensive management, the forest structure can be reduced and biodiversity can decline (Lonsdale et al., 2008). In many cases, this results in a reduced amount of deadwood, the essential resource for deadwood‐dependent (saproxylic) organisms (Müller & Bütler, 2010; Ódor et al., 2006). A diverse array of organisms including arthropods and fungi depend on deadwood. Deadwood is assumed to be important for 20%–30% of forest species in northern Europe (Stokland et al., 2012). Saproxylic arthropods fill various functional roles, from breaking down the deadwood substrate (xylophages and saprophages) to predating on other saproxylic organisms or parasitizing potential timber pest species (Ulyshen & Šobotník, 2018). Wood‐inhabiting fungi can have multiple interactions with other saproxylic organisms, e.g., allowing access to fresh deadwood by detoxifying plant defenses and/or by providing nutrition (Birkemoe et al., 2018). Several saproxylic insects are associated with wood‐decaying fungi (Biedermann & Vega, 2020) or even dependent on a particular fungal species for nutrition (Birkemoe et al., 2018; Floren et al., 2015; Jonsell & Nordlander, 2004). Together, these saproxylic organisms (invertebrates, fungi, bacteria and other microbes) accelerate the decay process of deadwood and are important for carbon sequestration, nutrient recycling, and overall biodiversity in the forests (Parisi et al., 2018; Stokland et al., 2012; Ulyshen, 2016).

Beside the amount of deadwood, the decay stage and deadwood quality are measures of resource availability for saproxylic organisms and suitable indicators for the biodiversity of arthropods (Lassauce et al., 2011; Müller & Bütler, 2010). Physical–chemical properties (e.g., lignin content, wood pH, moisture content) of deadwood (Hoppe et al., 2016), different decay stages, and the diameter of deadwood can affect the diversity of saproxylic organisms (Brin et al., 2011; Schiegg, 2001). In addition, saproxylic diversity is affected by local microclimatic factors such as temperature and humidity that are influenced by canopy openness and exposure to sunlight (Müller et al., 2020; Seibold et al., 2016).

Studies that assessed the impact of urbanization on arthropods along a rural–urban gradient yielded contrasting results. Some studies reported high abundances of several arthropod groups in urban areas (herbivorous arthropods: Raupp et al., 2010). Other studies, however, found a decline in the abundance and/or species richness of several arthropod groups from the rural surroundings to the city center (butterflies and moths: Merckx & Van Dyck, 2019; Diptera: Nelson & Forbes, 2014; Theodorou et al., 2020; carabid beetles: Niemelä & Kotze, 2009; Piano et al., 2020; parasitoid wasps: Fenoglio & Salvo, 2010; Nelson & Forbes, 2014; overall terrestrial arthropods: Piano et al., 2020). However, knowledge about the potential impact of urbanization on saproxylic organisms is scarce. Some studies investigated the microhabitat of tree hollows in veteran trees in urban parks and found a high diversity of saproxylic beetles in Helsinki (Peuhu et al., 2019) and the presence of endangered beetles (e.g., *Osmoderma eremita*) in Rome (Carpaneto et al., 2010). However, to our knowledge, no study investigated so far the potential effects of urbanization on the overall saproxylic insect community and fungi along a rural–urban gradient.

We used a standardized sampling procedure to examine whether the degree of urbanization, forest size, and/or local forest characteristics (including naturally occurring deadwood and branch characteristics) affected the communities of saproxylic insects and fungi. We exposed bundles consisting of freshly cut oak (*Quercus robur*) and beech (*Fagus sylvatica*) branches in 25 broad‐leaved forests along a rural–urban gradient in the city of Basel (Switzerland) and its surroundings for 8 months and subsequently collected the saproxylic insects in emergence traps for 10 months. In particular, our study aimed to assess: (1) the number of total saproxylic individuals (saproxylic beetles, flies, moths, and parasitic wasps) emerging from the deadwood bundles; (2) the taxonomic richness of beetles, flies, and moths; (3) the proportion of saproxylic individuals belonging to different ecological feeding guilds; (4) the proportion of the taxonomic richness of various feeding guilds; and (5) the taxon composition of all saproxylic insects that emerged, and in particular that of the beetles.

We also assessed whether (6) the number of fungal operational taxonomic units (OTUs) and (7) their composition on the bundles were influenced by the degree of urbanization, forest size, local forest characteristics, and branch characteristics. Finally, we examined whether (8) there exist fungal OTU–saproxylic insect associations on the bundles exposed.

We expected that an increase in the degree of urbanization and reduction in forest size both negatively affected the species richness and abundance of saproxylic insects because many species of these groups might be sensitive to forest fragmentation and habitat isolation, altered environmental conditions, and a reduced amount of deadwood in isolated urban forests (Lassauce et al., 2011; Schiegg, 2001). In contrast, we expected pioneer saproxylic beetles to be less affected by an increase in degree of urbanization and reduction in forest size because many of them are excellent flyers (e.g., bark beetles; Gossner et al., 2013; Komonen & Müller, 2018). Similar to related (albeit nonsaproxylic) insects like carabid beetles (Niemelä & Kotze, 2009), flies (Avondet et al., 2003), and moths (Piano et al., 2020), we expected the taxonomic composition to shift with urbanization. We also expected some local habitat variables such as resource availability (deadwood amount) to positively affect saproxylic individuals (Schiegg, 2001; Seibold et al., 2015) and that microclimatic variables (e.g., wood moisture content of the bundles exposed) influenced saproxylic insect richness and abundance (Vanderwel et al., 2006). Regarding saproxylic fungi, we expected a reduction in fungal OTUs with increasing degree of urbanization, decreasing forest size, and decreasing percentage forest area in the surroundings, because the microclimatic conditions are altered in small urban forest fragments; in particular, temperatures are higher and humidity lower (Bässler et al., 2010; New, 2015), and there is reduced amount of deadwood (Lonsdale et al., 2008). In turn, we expected a decrease in the proportion of mycetophagous and saprophagous insects with increasing degree of urbanization and decreasing forest size because of the reduced diversity of fungi or decayed wood material, which they feed on (Birkemoe et al., 2018; Vanderwel et al., 2006). Many saproxylic insects have strong preferences for specific deadwood‐decaying fungi (Biederman & Vega, 2020; Floren et al., 2015); therefore, we expected to find associations between taxonomic composition of insects and fungal OTUs.

## 2 MATERIALS AND METHODS

### 2.1 Study area

The study was carried out in the city of Basel and its surroundings (northwestern Switzerland; 47°34′N, 7°36′E, elevation: 245–565 m a.s.l.). There are approximately 200,000 residents living in Basel city with a population density around 5,000 inhabitants per km^2^ (www.bfs.admin.ch). The study area covers 88.3 km^2^, consisting of 43.6 km^2^ (49.4%) residential area, 16.1 km^2^ (18.3%) agricultural land, 25.5 km^2^ (28.8%) forest, 2.2 km^2^ (2.5%) water bodies, and 0.9 km^2^ (1.0%) other. In the study area, the total annual precipitation averages 842–1,005 mm and annual mean temperature 10.2–10.9°C (records from 1981 to 2010, www.meteoswiss.admin.ch).

To examine the potential effect of urbanization and forest size on saproxylic fauna, we chose 25 broad‐leaved forests that ranged in size from 0.1084 ha to 359.0 ha along an urbanization gradient (Table S1). The forests examined differ in their historical development and consequently in age. Twenty of them are surrounded by settlements and agricultural lands and are no longer connected to large continuous forests. Ten of these forests are remnants of former large continuous forests (fragments), and four forests were planted after 1884, while six forests are part of larger forests (>40 ha). The remaining five forests are situated in the rural surroundings and were part of large, beech‐dominated forests (>52.0 ha; Table S1; Figure S1). Management of the forests (time since last thinning and management intensity) was similar among the forests investigated. The most abundant tree species in these forests are European beech (*Fagus sylvatica*), sycamore (*Acer pseudoplatanus*), European oak (*Quercus robur*), ash (*Fraxinus excelsior*), hornbeam (*Carpinus betulus*), and Norway maple (*Acer platanoides*). The ground vegetation in the forests has a high richness of vernal geophytes including *Anemone nemorosa*, *Ranunculus ficaria*, *Polygonatum multiflorum,* and *Arum maculatum*.

In each forest, we selected an area dominated by deciduous trees (60%–90% of all tree individuals). This investigation area (hereafter forest site) was defined as the polygon determined by the six adult trees on which the bundles were attached (see below). The investigation area varied in size from 0.1 ha to 2.88 ha because some forests were extremely small and the distance between adult trees varied strongly among sites (Table S1). We assessed the degree of urbanization of each forest site, expressed as the percentage cover of sealed area within a radius of 500 m around the center of each forest site, using satellite images (Google Earth, 2009, 2020; Kanton Basel‐Landschaft, https://www.geoview.bl.ch/) and the pixel counting function of Adobe Photoshop (version 10.0.1). The degree of urbanization of the 25 forest sites examined ranged from 0% to 70% sealed area (Table S1).

### 2.2 Exposure of deadwood bundles in the field

To assess species richness and abundance of saproxylic insects, we used a slightly modified version of the method developed by Müller et al. (2015). In each forest site, we exposed six tree branch bundles as bait for saproxylic insects. The bundles were tied to a tree at a height of 1.5 m above ground (Figure S2). To avoid effects of heterogeneous microclimatic and weather conditions, all bundles were attached to the south side of a beech or oak tree located in closed canopy stands. The distance between the bundles ranged from 5 to 80 m. The bundles consisted of six freshly cut branches, three each of beech (*Fagus sylvatica*) and oak (*Quercus robur*) (branch length: 80 cm; diameter: 1.5–7.3 cm). In each forest, we exposed the bundles from April 2017 to January 2018. Due to vandalism, seven bundles could not be retrieved, resulting in a total of 143 bundles with 429 beech and 429 oak branches.

### 2.3 Extraction of saproxylic fauna

**FIGURE 1:**
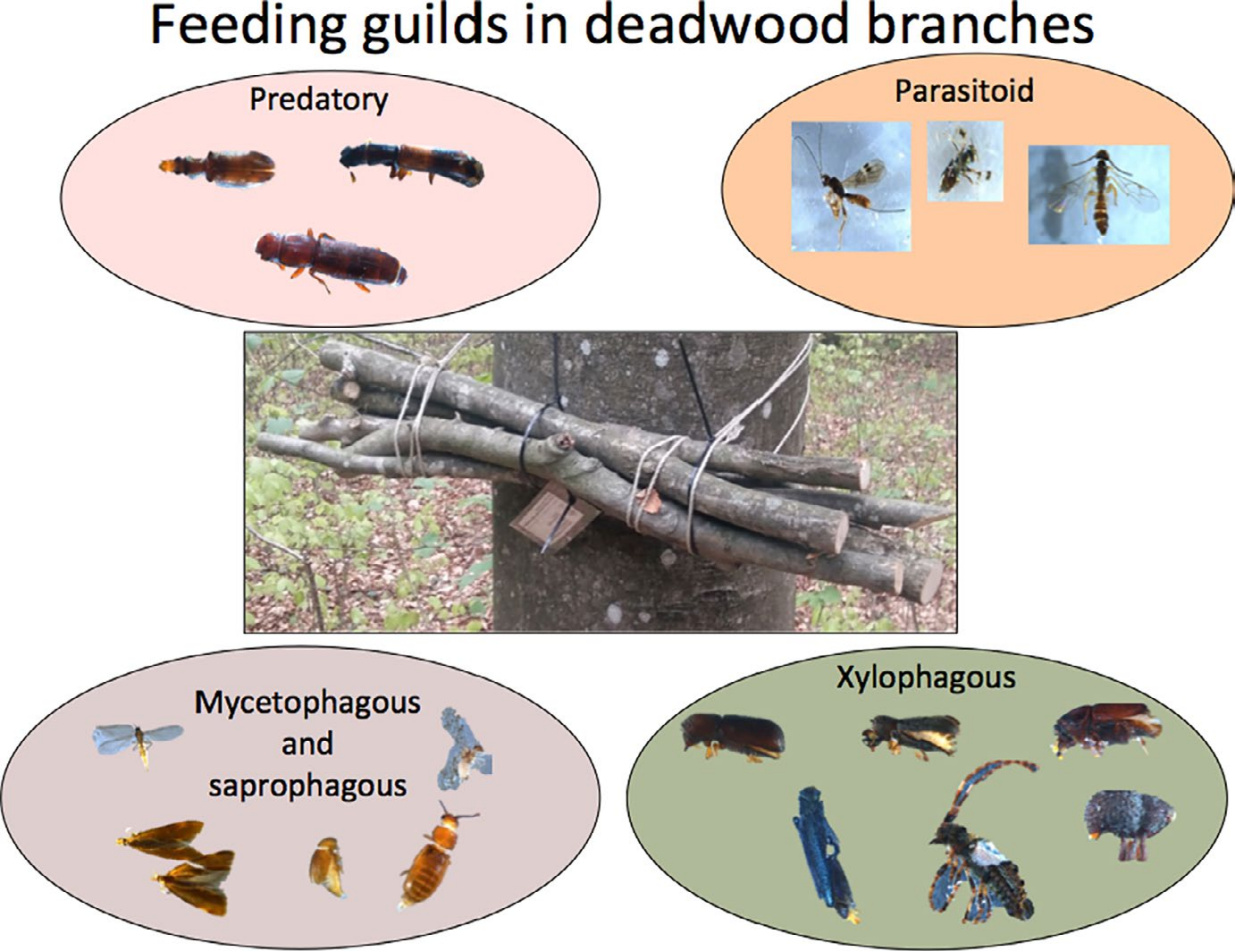
Graphical representation of the various ecological feeding guilds that occurred within the deadwood branches. Pictures of insects are of specimens that emerged from the branches

After an 8‐month exposure, we placed each bundle into a plastic tube (PVC; diameter 20 cm, length 100 cm) that acted as an emergence trap (Figure S2). The lids of the emergence traps had a hole (51 mm) covered with a fine mesh (0.5 mm) for ventilation (Figure S3). We placed the emergence traps in frames in an open shelter situated in a forest. Thus, the emergence traps were reared under natural daily temperature fluctuations (Figure S3). To avoid differences in the rearing conditions, we randomly changed the positions of the emergence traps in the frames every month. To examine whether the rearing conditions were similar in the emergence traps, we measured the temperature inside several traps using thermo buttons (Thermo button 21 G; software ThermoTrack PC Pro 17). We obtained the emerging saproxylic organisms from attached transparent collection containers filled with 70% ethanol monthly (except January) from February to end of October 2018 (Figure S3). The taxonomic resolution applied in various insect groups and the literature used for species determination are listed in Appendix S1, and a graphical representation of the saproxylic insects extracted is depicted in Figure 1.

### 2.4 Branch characteristics and fungal diversity (OTUs)

Macagno et al. (2015) showed that the size of a bundle can influence the colonization rate of saproxylic species. We therefore determined the volume of each bundle by measuring the diameter and length of the six branches. To assess fungal species diversity, we used subsamples of drilling chips from each branch. We used an electric drill with a bit diameter of 14 mm to obtain three drilling chip samples of each branch (at 1/4, 1/2 and 3/4 of its length) in December 2018. Within each bundle, we pooled the nine subsamples of the three beech and those of the three oak branches resulting in a total of 143 beech and 143 oak samples. The samples were weighed, freeze‐dried, and milled, and the resulting fine sawdust was used for the subsequent analyses. Wood moisture content was measured as loss during freeze‐drying, and the pH was assessed in distilled water (1:10). Lignin concentration was assessed with protein‐free sawdust using the acetyl bromide method (Moreira‐Vilar et al., 2014).

We extracted DNA from 200 mg of each sawdust sample using the Quick‐DNA Fecal/Soil Microbe Miniprep Kit (Zymo Research) following the manufacturer's instructions. DNA quantity and quality were measured using Nanodrop. We determined fungal OTUs using the F‐ARISA method with the fungal‐specific primer ITS1 (Gardes & Bruns, 1993) fluorescently labeled at the 5′‐end (FAM) and the unlabeled ITS4 (White et al., 1990). Polymerase chain reactions (PCR; 40 μl) consisted of 10 μl of template DNA (20–25 ng/μl), 8 μl Master Mix (5x FIREPOL Master Mix, Solis BioDyne), 1 μl Primer ITS1‐*F* (10 μM), 1 μl Primer ITS4 (10 μM), and 20 µl sterile water. Amplification was achieved in an Eppendorf Mastercycler Pro (Vaudaux‐Eppendorf AG) under the following conditions: initial 5 min heat activation step at 95°C, followed by 35 amplification cycles of denaturation at 95°C for 60 s, annealing at 55°C for 60 s, and extension at 72°C for 90 s, with a final extension step at 72°C for 7 min. All Polymerase chain reactions were done in duplicate. PCR products were cleaned‐up using the NucleoSpin gDNA Clean‐up kit (Macherey‐Nagel, Oensingen, Switzerland). Samples were prepared as GeneScan samples (1.5 μl DNA sample (20 ng/μl); 1.5 μl GeneScan 1200LIZ size standard; 17 μl Hi‐Di Formamide; Applied Biosystems, Life Technologies). Fragment analysis was conducted by Macrogen Inc. (Amsterdam, The Netherlands) by resolving the PCR products on a capillary sequencer (ABI 3730 XL DNA Analyzer). The size of the fluorescent amplified fragments was quantified using Peak Scanner software (version 1.0, Applied Biosystems, Inc.). Peaks with a size ranging from 390 to 1,000 bp were considered in the analyses (Ranjard et al., 2001), and according to Taggart et al. (2011), peak sizes obtained were rounded to the nearest base pair. To avoid possible background noise, only peaks with a signal above 1% of the sum of all peak areas were included in the analyses (Li et al., 2007). Peaks that differed in size by more than 2.0 bp were each considered as separate fungal OTU (Barto et al., 2011). The resulted OTUs of the pairs of the two PCR replicates were highly correlated (beech: *r_s_* = .78, *n* = 143, *p* < .0001; oak: *r_s_* = .74, *n* = 142, *p* < .0001).

### 2.5 Forest site characteristics

The amount of deadwood present in a forest is a key factor determining the species assemblage of saproxylic beetles (Müller & Bütler, 2010). We therefore assessed the amount of naturally occurring deadwood within a radius of 12.62 m (500 m^2^) around each of the trees on which bundles were exposed using two standardized sampling methods. First, we applied the fixed area sampling (FAS) for coarse woody debris (CWD; ≥7 cm diameter; ≥1 m in length) following the guidelines of Tinner et al. (2009) and calculated the CWD volume using the method of Ligot et al. (2012). Second, we applied the line intercept sampling (LIS) for the fine woody debris (FWD) using a slightly modified method of Böhl and Brändli (2007). The LIS consisted of three 10‐m‐long transect lines per tree with a bundle, with the bundle acting as starting point. We recorded all FWD with a diameter measuring between 1.5 and 7 cm and with a length of ≥1 m that crossed the transect line. Furthermore, we assessed the decomposition stage of the naturally occurring deadwood by using the “Swiss army knife technique”, which enables distinguishing five stages of increasing decomposition (Tinner et al., 2009).

Tree species differ in their suitability as host for saproxylic arthropods (Parisi et al., 2018; Stokland et al., 2012). We therefore assessed the tree species richness, the abundance of each species and the breast height diameter of each tree in the 500 m^2^ area around each focal tree in all 25 forest sites. The microclimate in a forest stand can also influence the saproxylic beetle composition (Müller et al., 2020; Seibold et al., 2016). We placed two temperature loggers (Thermo button 21 G) on two separate branch bundles in each forest site. Temperature was recorded hourly from late April until mid‐November 2017. For each forest site, we used the median of the hourly recorded temperature values of the two temperature loggers in the analyses.

### 2.6 Statistical analyses

We analyzed the data at the site level using the mean of the six bundles from each forest. Previous analyses revealed that the volume of the branch bundles and the thickness of the branches had no influence on the number of different groups of saproxylic insects that emerged and on the number of fungal OTUs. We used generalized linear models (GLM) with quasi‐Poisson‐distributed errors and log‐link function to test the potential effects of the degree of urbanization, forest size, forest site characteristics, and branch characteristics on the number of individuals of different taxonomic groups. Previous analyses revealed overdispersion when using the GLM models with Poisson‐distributed errors. Taxonomic richness of beetles, flies, and moths was analyzed separately with the same factors using GLM but with Poisson‐distributed errors.

We applied GLM with quasibinomial‐distributed errors and log‐link function (previous analyses revealed overdispersion when a binomial error distribution was used) to examine potential effects of the degree of urbanization, forest size, forest site characteristics, and branch characteristics on the proportion of individuals belonging to different feeding guilds (mycetophagous, saprophagous, xylophagous, predatory and parasitic). We also analyzed the proportion of beetle individuals belonging to different ecological feeding guilds with the same factors using GLM with binomial‐distributed errors.

Potential effects of landscape and forest characteristics on the total number of fungal OTUs on bundles and on the number of fungal OTUs on both oak and beech branches were analyzed using similar GLM models with Poisson‐distributed errors.

Because forest size and percentage forest area within a radius of 500 m were both intercorrelated with the degree of urbanization, we used the residuals of the relationship between these two variables and degree of urbanization for the GLM analyses (linear models: forest size and degree of urbanization, *t*_1,23_ = −6.14, *p* < .001; percentage forest within 500 m and degree of urbanization, *t*_1,23_ = −4.74, *p* < .001). Other forest site variables (temperature, number of living tree individuals, number of living tree species, distance to nearest beech tree, distance to nearest oak tree) were also intercorrelated and thus not considered in the analyses.

Partial redundancy analysis (pRDA) was used to determine effects of the degree of urbanization, forest size, forest, and bundle characteristics on composition of total saproxylic insect individuals and taxonomic composition of beetles (80 different species/genera; see Table 1) and on the composition of fungal OTUs on bundles. For composition of total saproxylic insect individuals, we ran pRDA analysis on two taxonomic levels: first on the level of families and second on the level of species/genera. In both analyses, we omitted singletons from the data file. Both analyses revealed very similar results. Therefore, we present only the findings of the species/genera analysis for total saproxylic insect individuals. In all cases, the “species matrices” were Hellinger‐transformed prior to the analysis. Model selection was conducted by forward step‐wise selection from a null model containing only degree of urbanization. In each step, the variables of the full model including forest size, forest area in the surrounding of 500 m and forest characteristics (fine woody debris volume, m^3^/ha), and branch bundle characteristics (wood pH, lignin and moisture content) that most significantly improved the model fit were added. This process continued until no further variable improved significantly the model fit (cut‐off *p* = .05). The final model was checked for variance inflation to detect collinearity of the variables included. Significant effects of the variables selected for the final model were tested using a post hoc ANOVA with 999 permutations. All pRDA analyses were conducted in R using the vegan package (Oksanen et al., 2017).

**TABLE 1:** Total number of saproxylic individuals and taxonomic units of various arthropod groups that emerged from the branch bundles

Taxonomic group	Identified to	Number of individuals		Taxonomic units	
		Total	Median (min–max)	Total	Median (min–max)
Beetles (Coleoptera)		37,591	1,501 (478–2,722)	—	—
Bark beetles (Scolytinae)	Species	17,655	639 (0–1,919)	5	3 (0–4)
Longhorn beetles (Cerambycidae)	Species	761	18 (0–110)	8	2 (0–4)
Jewel beetles (Buprestidae)	Species	1,461	36 (0–278)	4	2 (0–4)
Rove beetles (Staphylinidae)	Species	402	4 (0–157)	12	2 (0–6)
Minute brown fungus beetles (Latridiidae)	Species/Genus	335	3 (0–143)	5	1 (0–3)
Silken fungus beetles (Cryptophagidae)	Genus	11,641	468 (41–1,105)	2	2 (1–2)
Minute hooded beetles (Corylophidae)	Genus	4,535	92 (2–992)	2	1 (1–2)
Other beetles	Species/Genus	801	19 (2–228)	42	7 (2–15)
Flies (Diptera)		136,505	3,531 (1,413–18,836)	—	—
Dark‐winged fungus gnats (Sciaridae)	Family	88,230	1,872 (24–15,495)	1	1 (1–1)
Gall midges (Cecidomyiidae)	Subfamily	44,474	1,547 (781–4,156)	1	1 (1–1)
Fungus gnats (Mycetophilidae)	Family	466	1 (0–122)	1	1 (0–1)
Phorid flies (Phoridae)	Genus	181	4 (0–30)	1	1 (1–1)
Fruit flies (Drosophilidae)	Genus	91	2 (0–14)	1	1 (0–1)
Frit flies (Chloropidae)	Species	187	5 (0–28)	1	1 (0–1)
Dung midges (Scatopsidae)	Species	2,787	53 (0–471)	1	1 (0–1)
Hoverflies (Syrphidae)	Species	1	0 (0–1)	1	0 (0–1)
Craneflies (Tipulidae)	Family	1	0 (0–1)	1	0 (0–1)
Root maggot flies (Anthomyiidae)	Family	28	0 (0–26)	1	0 (0–1)
Soldier flies (Stratiomyidae)	Species	1	0 (0–1)	1	0 (0–1)
Long‐legged flies (Dolichopodidae)	Genus	56	0 (0–12)	1	0 (0–1)
Lauxanid flies (Lauxaniidae)	Species	2	0 (0–1)	1	0 (0–1)
Wasps (Hymenoptera)		11,268	43 (4–194)	—	—
Chalcid wasps (Chalcidoidea)	Superfamily	5,148	157 (49–898)	1	1 (1–1)
Braconid wasps (Braconidae)	Family	5,675	191 (1–602)	1	1 (1–1)
Ichneumonid wasps (Ichneumonidae)	Family	431	11 (0–107)	1	1 (0–1)
Wood wasps (Xiphydridae)	Family	14	0 (0–2)	1	0 (0–1)
Moths (Lepidoptera)		1,555	43 (4–194)	—	—
*Oecophora bractella* (Oecophoridae)	Species	30	0 (0–12)	1	0 (0–1)
*Metalampra italica* (Oecophoridae)	Species	1,299	33 (1–178)	1	1 (1–1)
*Shiffermuelleria schaefferella* (Oecophoridae)	Species	2	0 (0–1)	1	0 (0–1)
*Epicallima formosella* (Oecophoridae)	Species	23	0 (0–5)	1	0 (0–1)
*Nemapogon* spp. (Tineidae)	Genus	201	8 (0–18)	1	1 (0–1)
True bugs (Heteroptera)		216	5 (0–49)	—	—
*Xylocoris cursitans* (Anthocoridae)	Species	215	5 (0–49)	1	1 (0–1)
*Aneurus laevis* (Aradidae)	Species	1	0 (0–1)	1	0 (0–1)
Thrips (Thysanoptera)		72	1 (0–16)	—	—
*Xylaplothrips fulginosus*	Species	4	0 (0–1)	1	1 (0–2)
*Hoplothrips cortices*	Species	23	0 (0–16)	1	0 (0–1)
*Poecilothrips albopictus*	Species	44	0 (0–11)	1	0 (0–1)
*Aeolothrips versicolor*	Species	1	0 (0–1)	1	0 (0–1)
Barklice (Psocoptera)	Order	6,324	47 (12–2,865)	—	—
Lacewings (Neuroptera) larvae	Order	2	0 (0–1)	—	—
Snakeflies (Raphidioptera)	Order	1	0 (0–1)	—	—
Total saproxylic individuals		193,534	5,740 (2,773–20,578)	—	

Note

Median (minimum and maximum) values are shown for the 25 forest sites.

We used Co‐inertia Analysis (COIA; Dolédec & Chessl, 1994) to assess the potential association between fungal OTUs and saproxylic beetles. Co‐inertia analysis detects a common space into which the fungal OTUs and saproxylic insects can be projected and compared (Dolédec & Chessl, 1994). We first removed fungal OTUs and taxonomic units that were detected at only a single site to down weight the effects of rare species. Prior to COIA, principal component analysis (PCA) was performed on Hellinger‐transformed fungal OTUs and saproxylic insects community datasets. The strength of the coupling between each paired table was evaluated with the RV coefficient. This coefficient gives a measure of overall similarity of the two datasets and takes a value between 0 and 1: the closer the coefficient approaches to 1, the stronger the correlation between the two datasets. We then used Monte‐Carlo tests (with 999 random permutations) to assess the significance of the correlations. Co‐inertia analysis and Monte‐Carlo tests were performed with the “ade4” R package using the functions “coinertia” and “randtest”, respectively. Finally, we calculated the lengths of the arrows (Figure 7). We applied GLM models with Gaussian distribution and log‐linkage to test whether the degree of urbanization, forest size, and forest and bundle characteristics affect the fungal OTU–insect association (length of the arrows). All statistical analyses were carried out using the software R (R Core Team, 2015, version R 3.3.3 and version R 3.6.1).

## 3 RESULTS

### 3.1 Number of individuals and taxonomic richness of saproxylic insects

In total, 193,534 saproxylic insect individuals emerged from the branch bundles exposed in the 25 forest sites (Table 1). Beetles (Coleoptera) and flies (Diptera) were the two most abundant groups with 37,591 individuals (80 species/genera) and 136,505 individuals (13 different saproxylic fly families), respectively (Table 1). Among wasps (Hymenoptera), 11,268 individuals belonging to 4 families/superfamily emerged. Also, 1,555 individuals of saproxylic moths emerged (5 species/genera; Table 1). Other taxonomic groups (true bugs, thrips, snakeflies, and lacewings) were represented by just a few individuals (Table 1).

### 3.2 Factors influencing the number of saproxylic individuals

The 25 forest sites varied considerably in total number of saproxylic individuals that emerged from the six branch bundles (Table 1). Considering various taxonomic groups, the among‐site variation in number of saproxylic individuals was most pronounced in moths (49‐fold), followed by total flies (13‐fold), total wasps (12‐fold), and then beetles (sixfold; Table 1).

**FIGURE 2:**
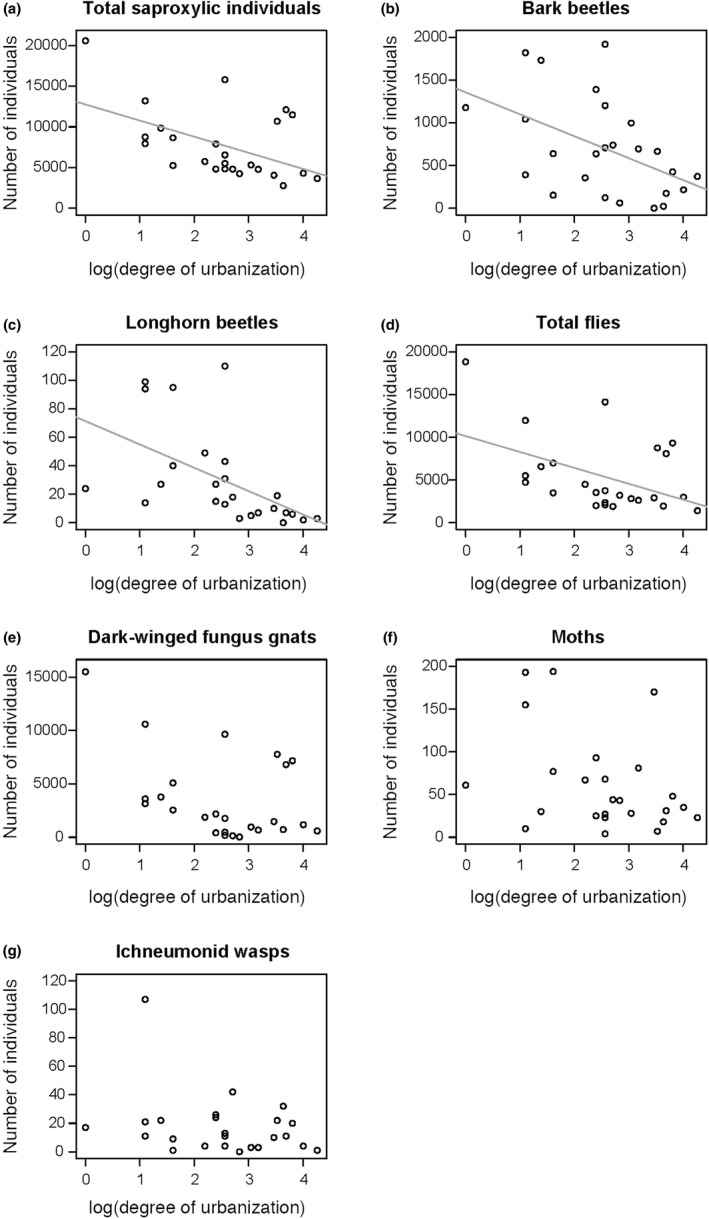
Effect of degree of urbanization on the number of (a) total saproxylic individuals, (b) bark beetles, (c) longhorn beetles, (d) total flies, (e) dark‐winged fungus gnats, (f) moths, and (g) ichneumonid wasps. Lines indicate a significant correlation between the degree of urbanization and the number of individuals of the different insect groups

The total number of saproxylic individuals decreased with increasing degree of urbanization (*F*_1,23_ = 8.85, *p* = .007; *r_s_* = −.48, *n* = 25, *p* = .016; Table S2; Figure 2a). Similarly, in several insect groups the number of individuals decreased with increasing degree of urbanization: in bark beetles (*F*_1,23_ = 7.20, *p* = .016; *r_s_* = −.47, *n* = 25, *p* = .019; Table S2; Figure 2b), longhorn beetles (*F*_1,23_ = 16.70, *p* < .001; *r_s_* = −.73, *n* = 25, *p* < .001; Table S2; Figure 2c), and total flies (*F*_1,23_ = 11.82, *p* = .003; *r_s_* = −.40, *n* = 25, *p* = .050; Table S2; Figure 2d). Dark‐winged fungus gnats were also affected by the degree of urbanization (*F*_1,23_ = 8.53, *p* = .009; Figure 1e), as were saproxylic moths (*F*_1,23_ = 4.89, *p* = .041; Table S2; Figure 2f) and ichneumonid wasps (*F*_1,23_ = 4.60, *p* = .047; Table S2; Figure 2g).

The numbers of jewel beetles (*F*_1,22_ = 8.07, *p* = .010) and chalcid wasps (*F*_1,22_ = 19.66, *p* < .001) were affected by forest size. Furthermore, several landscape and forest site characteristics influenced the number of saproxylic insects. The number of total flies (*F*_1,21_ = 4.57, *p* = .047; Table S2) was related to the percentage of forest within a radius of 500 m, while the number of dark‐winged fungus gnats (*F*_1,21_ = 4.30, *p* = .052; *r_s_* = .43, *n* = 25, *p* = .034; Table S2) was positively influenced by the percentage of forest within a radius of 500 m.

The numbers of longhorn beetles (*F*_1,21_ = 12.77, *p* = .002) and gall midges (*F*_1,21_ = 4.36, *p* = .050; Table S2) were affected by the volume of fine woody debris (Table S2). The number of total saproxylic beetles (*F*_1,21_ = 12.44, *p* = .002; *r_s_* = −.52, *n* = 25, *p* = .008; Table S2) was negatively affected by a more advanced stage of decomposition of the naturally occurring deadwood. The decomposition stage also influenced the numbers of jewel beetles (*F*_1,21_ = 4.64, *p* = .044; Table S2) and chalcid wasps (*F*_1,20_ = 18.93, *p* = .003; Table S2). The numbers of total flies (*F*_1,18_ = 4.88, *p* = .041; *r_s_* = .53, *n* = 25, *p* = .005; Table S2), dark‐winged fungus gnats (*F*_1,19_ = 4.82, *p* = .042; *r_s_* = .62, *n* = 25, *p* = .001; Table S2), other flies (other than the dominant fly families, dark‐winged fungus gnats and gall midges; *F*_1,18_ = 14.54, *p* = .001; *r_s_* = .63, *n* = 25, *p* < .001; Table S2), and saproxylic moths (*F*_1,17_ = 4.54, *p* = .048; *r_s_* = .51, *n* = 25, *p* = .010; Table S2) all increased with increasing wood moisture content of branches. The number of longhorn beetles was affected by wood pH of the branches (*F*_1,18_ = 4.48, *p* = .049; Table S2).

### 3.3 Taxonomic richness of saproxylic insects

Taxonomic richness of beetles, flies, and moths was not significantly influenced by the degree of urbanization, forest size, or any other forest site or branch characteristics (Table S3).

### 3.4 Number of individuals and taxonomic richness of ecological feeding guilds of saproxylic insects

The saproxylic individuals were assigned to five feeding guilds (mycetophagous, saprophagous, xylophagous, predatory and parasitoid; Table S4) in all taxonomic groups. In total, there were 147,493 mycetophagous individuals. Among them, we recorded 12,508 mycetophagous beetles belonging to 18 species (Table 2). We also recorded 133,358 mycetophagous fly individuals belonging to five families (Table 2).

**TABLE 2:** Number of individuals and taxonomic units belonging to different feeding guilds that emerged from the branch bundles exposed in the 25 forest sites

Taxonomic group	Number of individuals		Taxonomic units	
	Total	Median (min–max)	Total	Median (min–max)
Total mycetophagous individuals	147,493	4,143 (1,924–18,769)	32	13 (6–16)
Beetles	12,508	489 (48–1,146)	18	6 (2–8)
Flies	133,358	3,511 (1,411–18,659)	5	4 (2–4)
Dark‐winged fungus gnats (Sciaridae)	88,230	1,872 (24–15,495)	1	1 (1–1)
Gall midges (Cecidomyiidae)	44,474	1,547 (781–4,156)	1	1 (1–1)
Other mycetophagous Diptera	654	8 (0–125)	3	2 (0–2)
Moths (Oecophoridae and Tineidae)	1,555	43 (4–194)	5	3 (1–4)
Bark bugs (Aradidae)	1	0 (0–1)	1	0 (0–1)
Thrips (Thysanoptera)	71	1 (0–16)	3	1 (0–2)
Total saprophagous individuals	13,785	370 (52–2,996)	—	—
Beetles	4,552	92 (3–992)	3	1 (1–3)
Flies	2,909	83 (0–472)	5	2 (0–3)
Barklice (Psocoptera)	6,324	47 (12–2,865)	1	1 (1–1)
Total xylophagous individuals	19,857	682 (25–2,115)	—	—
Beetles	19,843	681 (25–2,113)	30	8 (4–14)
Bark beetles (Scolytinae)^a^	17,566	636 (0–1,918)	3	2 (0–3)
Longhorn beetles (Cerambycidae)	761	18 (0–110)	8	2 (0–4)
Jewel beetles (Buprestidae)	1,461	36 (0–278)	4	2 (0–4)
Snout beetles (Curculionidae)	27	1 (0–7)	6	1 (0–2)
Other beetles	28	1 (0–4)	9	1 (0–3)
Wood wasps (Xiphydridae)	14	0 (0–2)	1	0 (0–1)
Total predatory individuals	964	30 (4–180)	—	—
Beetles	688	17 (1–172)	29	6 (1–12)
Flies	57	1 (0–12)	2	1 (0–1)
Minute pirate bugs (Anthocoridae)	215	5 (0–49)	1	1 (0–1)
*Aeolothrips versicolor* (Thysanoptera)	1	0 (0–1)	1	0 (0–1)
Neuroptera larvae	2	0 (0–1)	1	0 (0–1)
Raphidioptera	1	0 (0–1)	1	0 (0–1)
Total parasitic wasps	11,254	454 (98–1,210)	—	—
Chalcid wasps (Chalcidoidea)	5,148	157 (49–898)	1	1 (1–1)
Braconid wasps (Braconidae)	5,675	191 (1–602)	1	1 (1–1)
Ichneumonid wasps (Ichneumonidae)	431	11 (0–107)	1	1 (0–1)

Note

Median (minimum and maximum) values of the forest sites are shown.

^a^ Excluding Ambrosia bark beetles which were categorized within the mycetophagous beetles.

We found 13,785 saprophagous individuals. Among them, there were 4,552 saprophagous beetle individuals comprising of three species. Regarding saprophagous flies, there were 2,909 individuals comprising of five families (Table 2). A total of 19,857 xylophagous individuals were recorded. The majority of them were xylophagous beetles (19,843 individuals) comprising of 30 species (Table 2). We found 964 predatory individuals. Among them, there were 688 predatory beetles belonging to 29 predatory species (Table 2). There were 11,254 parasitic wasp individuals (Chalcidoidea, Braconidae, and Ichneumonidae combined) belonging to three families/superfamily (Table 2). We also found 181 individuals of Phoridae flies (genus *Megaselia*) that could not be assigned to a functional group. These individuals were added to the total saproxylic individuals (Table 2), but not further considered in the analyses.

### 3.5 Factors influencing the proportion of individuals belonging to different feeding guilds

**FIGURE 3:**
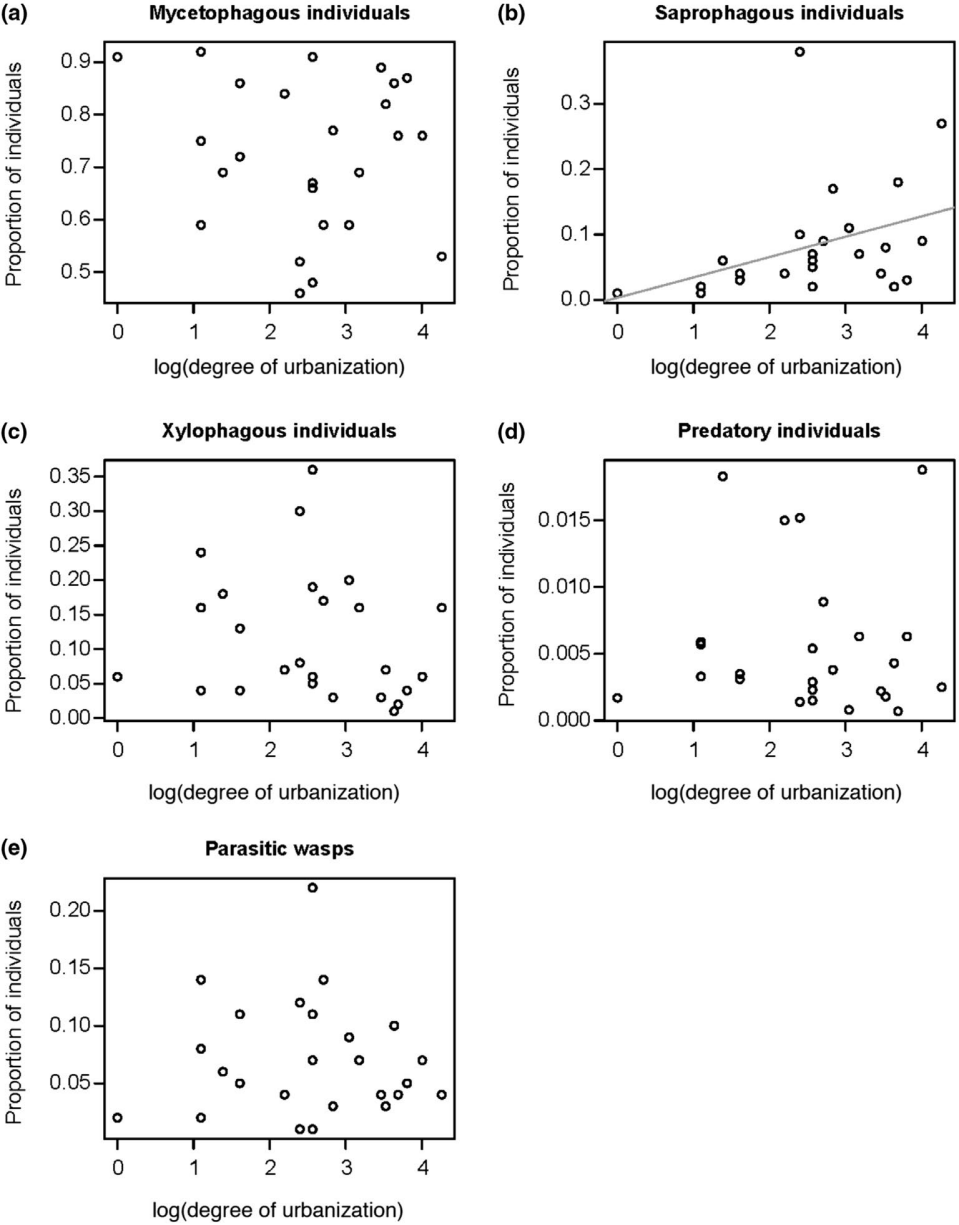
Effect of degree of urbanization on the proportion of (a) total mycetophagous, (b) saprophagous, (c) xylophagous, (d) predatory, and (e) parasitic wasp individuals

Among the feeding guilds, the proportion of saprophagous individuals increased with increasing degree of urbanization (*F*_1,23_ = 24.48, *p* < .001; *r_s_* = .53, *n* = 25, *p* = .006; Table S5; Figure 3b). In contrast, the proportion of saprophagous individuals decreased with increasing forest size (*F*_1,22_ = 18.47, *p* < .001; *r_s_* = −.40, *n* = 25, *p* = .046; Table S5) and was affected by the percentage of forest within a radius of 500 m (*F*_1,21_ = 8.25, *p* = .011; Table S5). Furthermore, the proportion of saprophagous individuals was negatively related to the stage of decomposition of the naturally occurring deadwood (*F*_1,19_ = 10.81, *p* = .004; *r_s_* = −.44, *n* = 25, *p* = .029; Table S5) and decreased with increasing wood moisture content (*F*_1,17_ = 9.35, *p* = .007; *r_s_* = −.68, *n* = 25, *p* < .001; Table S5). In contrast, the proportions of individuals belonging to the other feeding guilds (mycetophagous, xylophagous, predatory, and parasitoid) were neither influenced by the degree of urbanization nor by the size of the forests (Table S5; Figure 3). However, the proportion of mycetophagous individuals was affected by the percentage of forest within a radius of 500 m (*F*_1,21_ = 6.12, *p* = .025; Table S5) and was positively related to the stage of decomposition of naturally occurring deadwood (*F*_1,18_ = 16.99, *p* < .001; *r_s_* = .48, *n* = 25, *p* = .015; Table S5). Furthermore, the proportion of mycetophagous individuals increased with increasing wood moisture content of the branches (*F*_1,17_ = 12.52, *p* = .003; *r_s_* = .69, *n* = 25, *p* < .001; Table S5). Considering parasitic wasps, their proportion was only affected by the decomposition stages of naturally occurring deadwood (*F*_1,20_ = 6.36, *p* = .020; Table S5).

### 3.6 Proportion of taxonomic richness of ecological feeding guilds

The proportion of taxonomic richness of total mycetophagous, saprophagous, xylophagous, and predatory individuals and the taxonomic richness of mycetophagous, saprophagous, xylophagous, and predatory beetles were not significantly influenced by either the degree of urbanization, forest size, or any other forest site or branch characteristics (Table S6).

### 3.7 Total insect taxonomic composition and composition of beetles

Partial redundancy analyses revealed that the composition of total saproxylic insect individuals was neither altered by the degree of urbanization nor by forest size. The permutation analysis for the pRDA analysis showed that the wood moisture content of the branches (*F*_1,19_ = 6.00, *p* = .001) significantly changed the composition of total saproxylic insect individuals. Furthermore, the percentage of forest area within 500 m tended to affect the composition of total saproxylic insect individuals (*F*_1,19_ = 2.29, *p* = .053).

**FIGURE 4:**
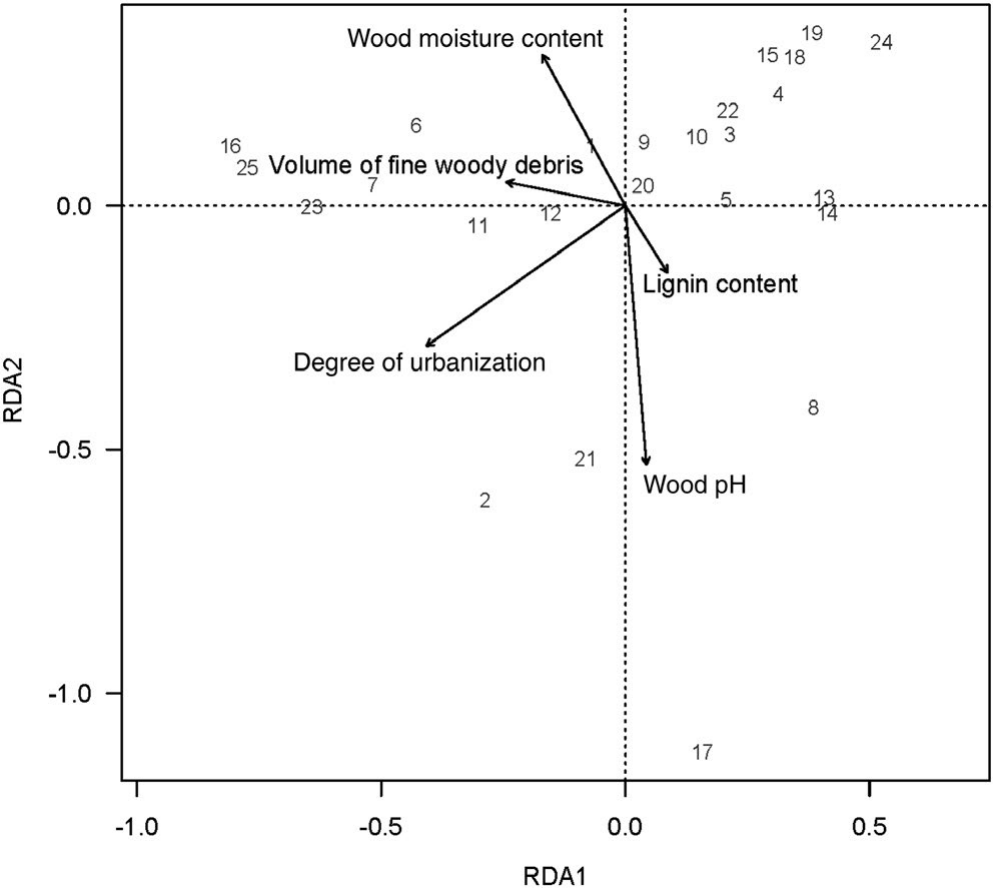
Results of constrained ordination analysis (RDA) showing the relationship of the taxonomic composition of beetles that emerged from the branch bundles exposed in 25 forests to the degree of urbanization and forest characteristics such as volume of fine woody debris and branch bundle characteristics including wood pH, wood moisture and lignin content (only significant variables are shown)

Considering saproxylic beetles, both the degree of urbanization (F_1,19_ = 5.92, *p* = .001) and the wood pH of the branches (*F*_1,19_ = 2.66, *p* = .025) altered the taxonomic composition of the beetles (Figure 4). Furthermore, the volume of fine woody debris (*F*_1,19_ = 2.11, *p* = .063) and the lignin content of the branches (*F*_1,19_ = 2.10, *p* = .070) tended to change the taxonomic composition of the beetles (Figure 4). The permutation test further revealed that these factors significantly separated the taxonomic composition of the beetles along the first (*F*_1,19_ = 6.39, *p* = .003) and second axis (*F*_1,19_ = 5.64, *p* = .003; Figure 4).

### 3.8 Diversity and composition of fungal species (OTUs)

Out of a total of 97 fungal OTUs that were recorded in the branch bundles exposed in the 25 forest sites, 90 fungal OTUs were found on beech branches and 91 fungal OTUs on oak branches. Total number of fungal OTUs ranged from 32 to 63 across the forest sites (median: 47 OTUs). The corresponding figures for the number of fungal OTUs found on beech branches were 26–44 (34) and 20–52 (34) for oak branches.

**FIGURE 5:**
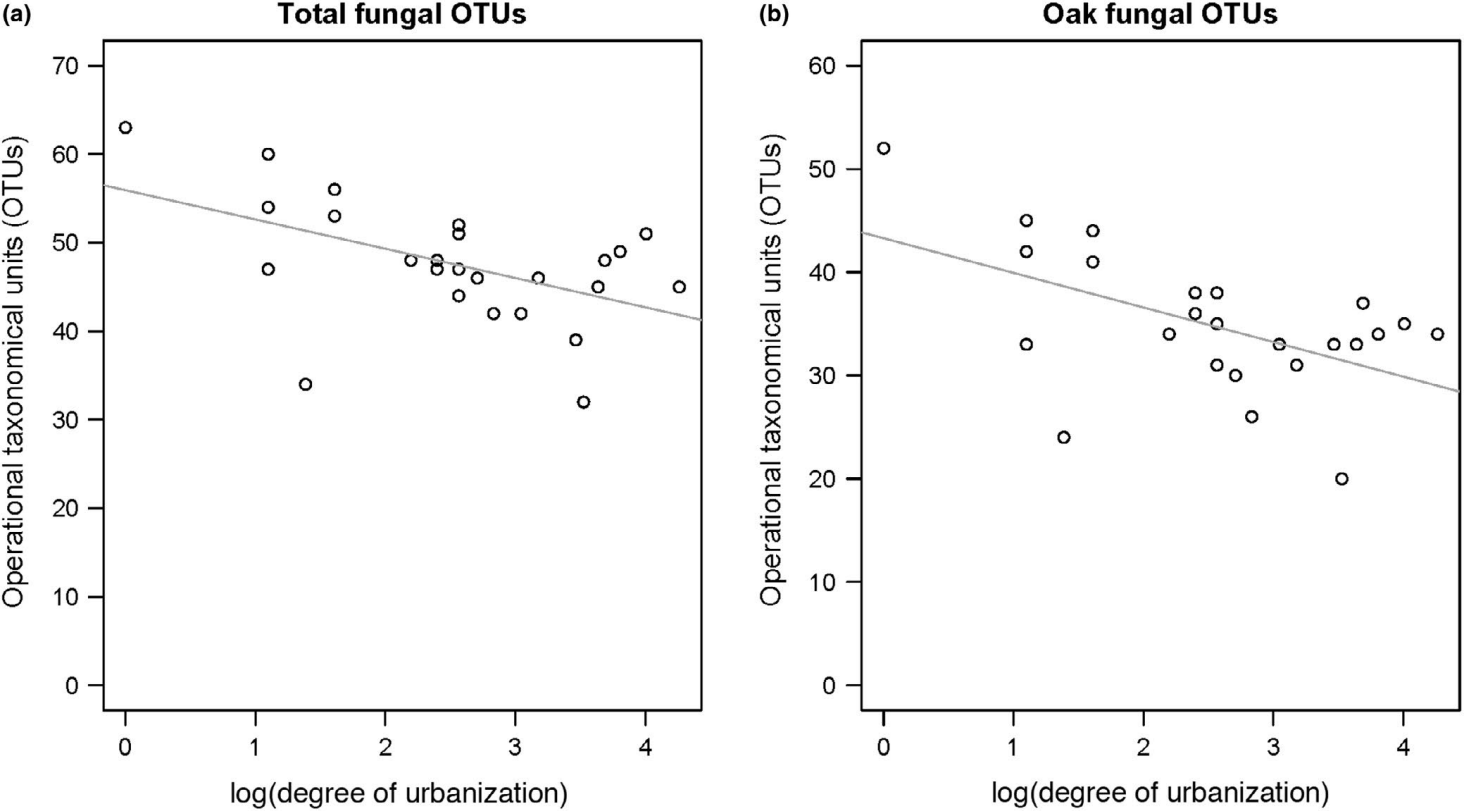
Effect of degree of urbanization on (a) the total number of fungal OTUs found on the branch bundles and (b) the number of fungal OTUs only found on oak branches

Total number fungal OTUs found on the entire bundles decreased with increasing degree of urbanization (χ^2^_1,23_ = 6.31, *p* = .012; *r_s_* = −.46, *n* = 25, *p* = .020). Similarly, the number of fungal OTUs found exclusively on oak branches decreased with increasing degree of urbanization (χ^2^_1,23_ = 8.84, *p* = .003; *r_s_* = −.41, *n* = 25, *p* = .042, Table S7; Figure 5a, b). In contrast, the number of fungal OTUs on beech branches was not affected by the degree of urbanization (Table S7). Overall, the size of the forests did not influence the number of total fungal OTUs as well as the number of fungal OTUs on beech and oak branches (all *p* > .20; Table S7). Considering forest site characteristics, both the total number of fungal OTUs and those found exclusively on oak branches were positively influenced by the volume of fine woody debris (total: χ^2^_1,20_ = 4.21, *p* = .040; *r_s_* = .45, *n* = 25, *p* = .025; oak: χ^2^_1,20_ = 4.91, *p* = .027; *r_s_* = .45, *n* = 25, *p* = .023; Table S7).

**FIGURE 6:**
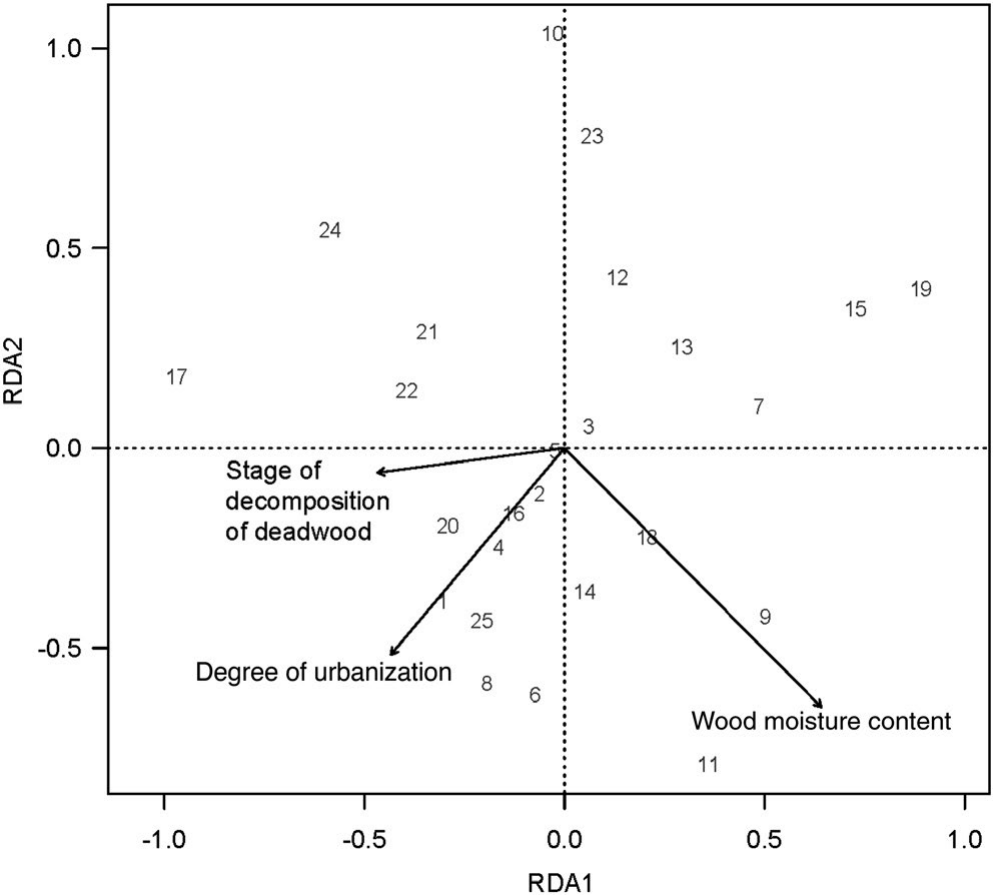
Results of constrained ordination analysis (RDA) showing the relationship of the taxonomic composition of fungal OTUs detected on branch bundles exposed in 25 forests to the degree of urbanization, stage of decomposition of naturally occurring deadwood in the forest sites and wood moisture content of the branch bundles exposed (only significant variables are shown)

Partial RDA showed that the degree of urbanization altered the composition of total fungal OTUs (Permutation test: *F*_1,21_ = 1.46, *p* = .048; Figure 6). Furthermore, decomposition stage of the naturally occurring deadwood (*F*_1,21_ = 1.45, *p* = .050) and the wood moisture content of the branches (*F*_1,21_ = 1.42, *p* = .072) affected the composition of total fungal OTUs. Considering the fungal OTUs found on beech branches, their composition was affected by the wood pH of the beech branches (*F*_1,23_ = 2.19, *p* = .033). Wood pH also separated fungal OTU composition on beech branches along the first RDA axis (*F*_1,23_ = 2.19, *p* = .027). In contrast, fungal OTU composition on oak branches was influenced both by the degree of urbanization (*F*_1,22_ = 2.14, *p* = .029) and wood pH of branches (*F*_1,22_ = 2.25, *p* = .014). Furthermore, the degree of urbanization separated the composition of the fungal OTUs along the first RDA axis (*F*_1,22_ = 2.99, *p* = .014).

The compositions of fungal OTUs found on either beech or oak branches were not associated (*RV* = 0.29, *p* = .465).

### 3.9 Association between total fungal OTUs and total saproxylic insects

**FIGURE 7:**
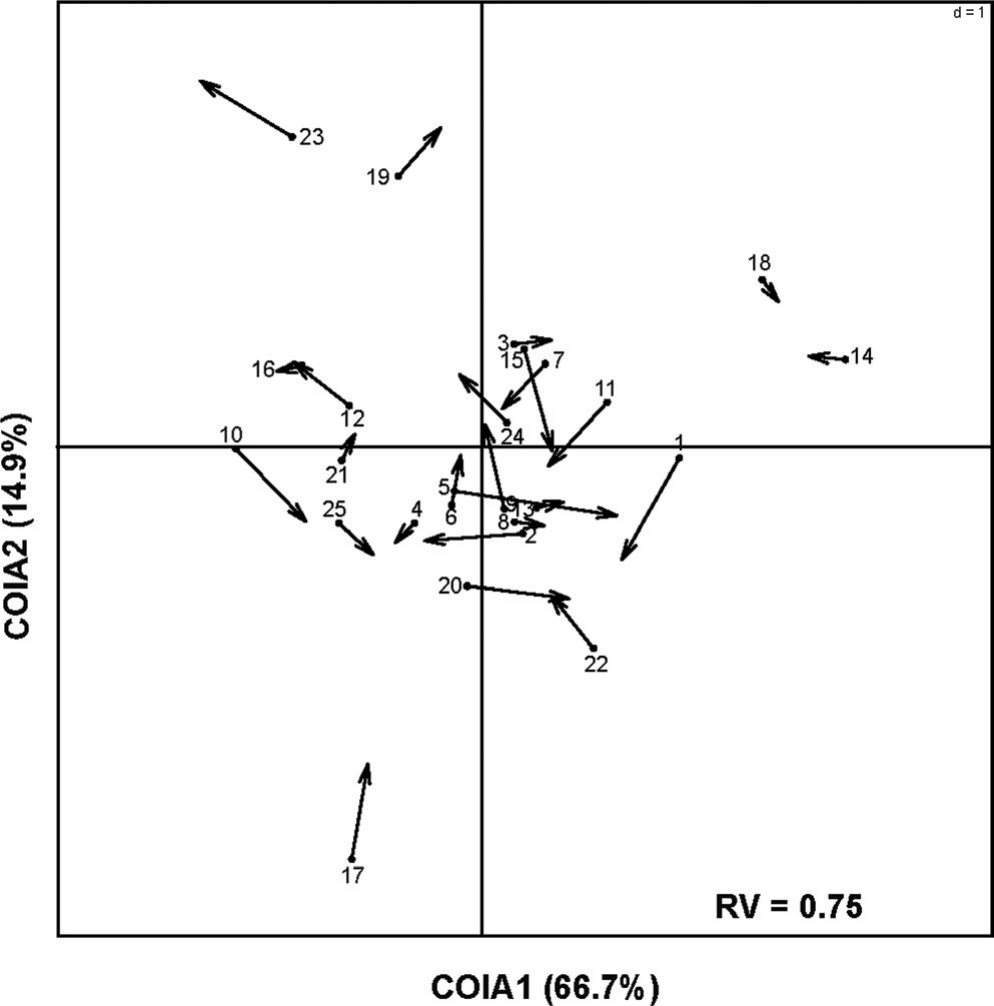
Co‐inertia analysis (COIA) between the compositions of total fungal OTUs and the total saproxylic insect individuals. The arrows link the co‐inertia scores resulting from the fungal OTUs data set to that obtained from the saproxylic insect data set. Circles locate sites as ordinated by fungal OTU composition, whereas arrows indicate the ordination of saproxylic insects

Co‐inertia analysis revealed a significant association between the composition of total fungal OTUs and that of the total saproxylic insect individuals *(RV* = 0.75, *p* = .005; Figure 7). The first two axes accounted for 80.4% of the total variance in fungi–saproxylic insects comparison (Figure 7).

The lengths of the arrows shown in Figure 7 illustrate the concordance between the two datasets. The shorter the arrows, the better the concordance between the composition of fungal OTUs and that of total saproxylic insects. Generalized linear model analysis revealed that the lengths of the arrows were exclusively related to the degree of urbanization (*F*_1,23_ = 4.98, *p* = .036), indicating that the association between OTU composition of fungi and that of saproxylic insects altered along the rural–urban gradient.

## 4 DISCUSSION

### 4.1 Effects of degree of urbanization on saproxylic individuals

Our study showed that the abundance of several groups of saproxylic insects including bark beetles, longhorn beetles, and total flies decreased in forests situated in increasingly urbanized areas, while forest size and local forest characteristics appeared to have a minor influence on the abundance of these insect groups. Furthermore, the total number of fungal OTUs (a potential proxy for fungal species richness) decreased with increasing degree of urbanization in the surroundings of the forests examined and was positively influenced by the volume of fine woody debris in the forest site. Thus, our study provides evidence that urbanization negatively impacts saproxylic insects and fungi.

Understanding how landscape characteristics affect biodiversity patterns at local and landscape scales is important for promoting biodiversity in urban environments (Beninde et al., 2015). In our study, the abundance of total saproxylic individuals was negatively related to the degree of urbanization. This finding is in line with the results of a recent study conducted in Belgian cities (Piano et al., 2020) and a global meta‐analysis showing that terrestrial arthropods were less abundant and diverse in more urbanized landscapes (Fenoglio et al., 2020). In contrast to these studies, we did not record any effect of degree of urbanization on the composition of total saproxylic insect individuals.

In our study, flies were the most abundant taxonomic group comprising of 71% of all saproxylic individuals. The observed negative effect of urbanization on total number of saproxylic individuals mainly resulted from a reduced number of flies recorded in forests situated in highly urbanized areas (Figure 2). Similar decreases in the abundance and/or richness of several fly families in urban areas were reported in other studies (total flies: Theodorou et al., 2020; Drosophilidae flies: Gottschalk et al., 2007; Chloropidae: Kozlov & Zvereva, 1997 and phorid flies: Durska, 1981). However, a high habitat quality (abundance of floral resources) in urban areas can support a high fly richness (Theodorou et al., 2020). Regarding saproxylic flies, no study assessed so far the effect of urbanization on this insect group.

Considering saproxylic moths, the number of individuals declined with increasing degree of urbanization, similar to the pattern found in the meta‐analysis of Fenoglio et al. (2020). Piano et al. (2020) also demonstrated that with increasing degree of urbanization macromoths became less diverse. Furthermore, several saproxylic moths had lower abundances in forest fragments than in large continuous old‐growth forests (Komonen et al., 2000). In urban environments, light pollution may disrupt movement of nocturnal moths between habitat patches and contribute to their decline (Bates et al., 2014). However, a study in Basel found that moths in urban areas exposed to strong light pollution (near streetlights) have a reduced flight‐to‐light behavior compared to those in rural populations (Altermatt & Ebert, 2016). The altered flight‐to‐light behavior may allow moth populations to persist in an urban setting.

Parasitoids (including the three wasp families/superfamilies recorded in our study) are an integral part of the saproxylic food web (Hilszczański, 2018). Many parasitoids are characterized by low abundances (Fenoglio & Salvo, 2010). It has therefore been suggested that parasitoids are more negatively affected by habitat fragmentation than their hosts (Tscharntke & Brandl, 2004). This has partly been confirmed as some parasitoids, including ichneumonid wasps, were found in lower abundances, species richness, and parasitism rates with increasing degree of urbanization (Fenoglio & Salvo, 2010). Similarly, we found a lower abundance of ichneumonid wasps in isolated urban forests than in rural forests (Figure 2), which might reduce the natural enemy regulation ecosystem service they provide (Fenoglio et al., 2020; Nelson & Forbes, 2014).

As we expected, total abundance and richness of saproxylic beetles were not affected by the degree of urbanization. In terms of species richness of saproxylic beetles, the importance of deadwood becomes more prominent with increasing spatial scales (Franc et al., 2007). However, the abundance of bark beetles comprising 47% of the saproxylic beetles and that of the longhorn beetles (2%) decreased with increasing degree of urbanization (Figure 2). As pioneer saproxylics, bark beetles colonize the early decay stage of deadwood (Parisi et al., 2018). Many bark beetle species are good dispersers and can find fresh deadwood substrates several kilometers away from their starting location (Komonen & Müller, 2018), even within an urban setting (Piel et al., 2005). However, using a rural–urban gradient approach, Piel at al. (2005) showed that the spruce bark beetle (*Ips typographus*) reached the highest abundance in areas with a moderate level of urbanization and that the abundance of bark beetles in the city center was negatively influenced by building density. In our study, the reduced abundance of beech bark beetles (*Taphrorychus bicolor*) in highly urbanized forests could be the result of dispersal barriers in urban areas and the difficulty in locating viable beech and oak hosts via volatiles or aggregation pheromones (Piel et al., 2005).

We also recorded a shift in total beetle species composition along the urbanization gradient. This result parallels the findings of some studies showing that urbanization changes the species composition of nonsaproxylic carabid beetles (Niemelä & Kotze, 2009) and that of staphylind beetles (Magura et al., 2013) and of microarthropod litter communities in urban forests (Malloch et al., 2020), and thus may reduce the functional diversity of the beetle community (Hagge et al., 2019).

### 4.2 Effects of forest size and local forest characteristics

Forest size and local forest characteristics including the amount of deadwood, its decay stage and moisture content have the potential to influence the abundance, species richness, and composition of saproxylic insects (Hagge et al., 2019; Lassauce et al., 2011; Seibold et al., 2015). In our study, forest size did not affect the abundance and species richness of most taxonomic groups with the exception of jewel beetles and chalcid wasps. Grohmann et al. (2003) also found no effect of forest size on the abundance of saproxylic beetles. This might frequently be the case for pioneer saproxylic species as investigated in our study. Pioneer saproxylic species often prefer single trees, forest edge habitats, and forests with an open canopy (Bouget et al., 2013; Müller et al., 2015; Vodka et al., 2009). The dominant bark beetles in our study (*T. bicolor* and *S. intricatus*) occur at sunny forest edges as well as in shaded forest interiors (Kappes & Topp, 2004; Véle & Horák, 2018; Wermelinger et al., 2007), and thus, no forest size‐abundance relationship can be expected.

Urban broad‐leaved forests are regularly disturbed by recreational activities and frequently by intensive forest management, which can reduce the amount of deadwood, the essential resource for deadwood‐dependent organisms (Müller & Bütler, 2010; Ódor et al., 2006). In our study, only the abundances of longhorn beetles, gall midges, and ichneumonid wasps were influenced by the volume of fine woody debris, but not by the volume of coarse woody debris. The majority of studies on saproxylics investigated the role of the amount of CWD and found a positive effect on the abundance and species richness of saproxylic insects (Lassauce et al., 2011; Seibold et al., 2015; Vanderwel et al., 2006). Most saproxylics show a preference for a certain deadwood diameter range (Brin et al., 2011; Schiegg, 2001); thus, the saproxylics emerging from the branches in our study responded more to FWD than to CWD in the surroundings. Vodka et al. (2009) reported a weak relationship between FWD and the abundance of saproxylic insects. Pioneer saproxylics in fine woody debris as investigated in our study are less sensitive to disturbance or changes in specific local habitat characteristics and may therefore respond on a larger landscape scale (Franc et al., 2007; Gossner et al., 2013; Økland et al., 1996; Schiegg, 2001). Apart from ichneumonid wasps, the other parasitic wasps may have great dispersal capabilities similar to their primary saproxylic beetle hosts and are therefore not bound to local deadwood resources (Gibb et al., 2008).

The decay stage of deadwood determines the abundance and species richness of saproxylics such as beetles, flies, and parasitic wasps (Gossner et al., 2013; Vanderwel et al., 2006). In our study, we considered the early decay stages of beech and oak branches and thus early colonizers on deadwood (Vanderwel et al., 2006). We only found an effect of the decay stages on the abundance of jewel beetles and chalcid wasps. Furthermore, the quality of deadwood can influence the abundance and species richness of saproxylic insects (Lassauce et al., 2011). In accordance with other studies (Hibbert, 2010; Hövemeyer & Schauermann, 2003; Irmler et al., 1996), we found that moisture content of the branches affected the abundance of flies and the proportion of mycetophagous individuals. Higher moisture content of branches provides ideal microclimatic conditions for the proliferation of deadwood fungi (Bässler et al., 2010), which in turn promotes mycetophagous flies, especially the dark‐winged fungus gnats with short generation length (Vanderwel et al., 2006). In addition, higher moisture content caused a significant shift in composition of total saproxylic insect individuals, probably by providing more ideal microclimatic conditions for mycetophagous beetles, flies, and moths (Thorn et al., 2018; Vanderwel et al., 2006). The shift in beetle species composition as a result of changed pH and lignin content of the dead wood branches could be caused by differences in fungal enzyme activity (Baldrian et al., 2016) and thus available decayed organic material for certain beetles to feed on.

### 4.3 Effects of degree of urbanization and local forest characteristics on saproxylic fungi

Saproxylic fungi are key players in the breakdown of deadwood and provide food resources for saproxylic insects (Birkemoe et al., 2018). We found a negative effect of the degree of urbanization on both the total number of OTUs and the number fungal OTUs exclusively occurring on oak branches. The reduction in number of fungal OTUs may be caused by the fragmentation of the forests and associated changes in microclimatic conditions (lower humidity). Furthermore, the lower number of fungal OTUs in forests situated in highly urbanized areas could be a result of limited dispersal of spores in fragmented forests (Kauserud et al., 2005; Küffer & Senn‐Irelt, 2005; Siitonen et al., 2005). Although fungal spores can be transported over large distances by wind and small animals (Komonen & Müller, 2018), fragmentation‐reduced spore dispersal may result in a decline in the re‐colonization of fungi species, especially in specialized fungi (Lonsdale et al., 2008). The microclimatic conditions in a forest affect fungal growth and fruiting, particularly in fungi growing on fine woody debris (Bässler et al., 2010). This might partly explain the lower fungal richness in forests in urban areas with a high temperature. In contrast to another study, we found no effect of forest size on the number of fungal OTUs (Küffer & Senn‐Irlet, 2005).

The positive impact of increasing volume of fine woody debris on total OTUs and fungal OTUs on oak branches indicates that the kind of forest management is important for promoting saproxylic fungi. We observed piles of branches left after logging in the forests. In the short to mid‐term (5–10 years), the amount of fine woody debris can increase the number of common fungi species (Pasanen et al., 2014) and might be important for the preservation of rare fungal species in intensively managed forests (Küffer & Senn‐Irlet, 2005; Nordén et al., 2004).

The degree of urbanization also caused shifts in the composition of fungal OTUs. Additionally, as reported in previous studies (Hoppe et al., 2016), physical–chemical properties of branches (wood pH and moisture) influenced the species composition of saproxylic fungi.

### 4.4 Effect of degree of urbanization on the proportion of mycetophagous and saprophagous insects

Surprisingly, the proportion of mycetophagous individuals was neither affected by the degree of urbanization nor by forest size. A previous study found a lower number of forest‐dwelling (mainly saprophilous and mycetophilous) staphylinid species in urban areas, probably because of increased dryness and reduction in organic matter on the forest floor (Magura et al., 2013). The fact that we actually found a higher proportion of saprophagous individuals in urban areas might be a result of the dominance of the cosmopolitan beetle *Sericoderus lateralis* (Corylophidae) that can reproduce asexually and thrives in cities around the world (Bowestead, 1999).

### 4.5 Insect–fungi association

In pieces of deadwood, fungi breakdown lignin and cellulose and detoxify secondary chemicals such as phenols, thus allowing access for saproxylic insects (Biedermann & Vega, 2020). Previous studies found that saprophagous and mycetophagous insects were associated with saproxylic fungi and that species richness of saproxylic insects increased with increasing fungal richness (Floren et al., 2015; Persiani et al., 2010). In accordance with these studies, we found significant associations between fungal OTUs and saproxylic insects. Urbanization negatively affected some saproxylic groups, which may disturb the saproxylic community network. In our study, the altered species composition of fungal OTUs with increasing degree of urbanization might have changed the interactions between saproxylic insect species and fungal OTUs.

## 5 CONCLUSIONS

Our study showed that alterations in abundances of saproxylic insects and the number of fungal OTUs along a rural–urban gradient could be driven by urbanization, and also partly by local characteristics (e.g., deadwood amount) and branch characteristics (wood moisture). This ultimately led to changes in taxonomic composition and altered linkages between saproxylic insect and fungi in urban areas, which in turn may influence the ecosystem functions of wood decomposition and nutrient recycling in urban forests.

Forest management could promote saproxylic insect and fungal diversity by increasing the amount of deadwood of various diameters including FWD in urban forests. Urban forests play an essential role for recreational activities. For security reasons this fact has to be taken into account when promoting standing deadwood (Fröhlich & Ciach, 2020). An alternative measure but not a real substitute for standing deadwood and large deadwood logs could be assembling and providing piles of branches that may serve as a refuge for some saproxylic insects (Jonsell et al., 2007) and fungi (Pasanen et al., 2014).

## CONFLICT OF INTEREST

The authors declare no conflict of interest.

## AUTHOR CONTRIBUTION

**Sandro Meyer:** Conceptualization (lead); Data curation (lead); Formal analysis (equal); Funding acquisition (equal); Methodology (equal); Visualization (lead); Writing‐original draft (lead); Writing‐review & editing (lead). **Hans‐Peter Rusterholz:** Conceptualization (equal); Data curation (supporting); Formal analysis (equal); Investigation (supporting); Methodology (supporting); Project administration (supporting); Supervision (equal); Writing‐review & editing (supporting). **Bruno Baur:** Conceptualization (equal); Data curation (supporting); Formal analysis (supporting); Funding acquisition (equal); Investigation (supporting); Methodology (equal); Project administration (equal); Resources (lead); Software (lead); Supervision (equal); Validation (lead); Writing‐review & editing (equal).

## Supporting information

Appendix S1

## Data Availability

The abundance data of the saproxylic insects, the number of fungal OTUs, the taxonomic richness, and proportion of insects belonging to different feeding guilds, as well as the local forest site and landscape characteristics and branch characteristics, are available from the Dryad Digital Repository (https://doi.org/10.5061/dryad.gf1vhhmnn).
