# Supplementary material for: Saproxylic insects and fungi in deciduous forests along a rural–urban gradient
Source: Ecol Evol. 2021 Jan 27;11(4):1634–52. doi: 10.1002/ece3.7152 (PMC7882972; doi:10.1002/ece3.7152)
Supplement: Supplementary file 1 — Appendix S1 [file ECE3-11-1634-s001.docx]

**Appendix S1. Taxonomical resolution and literature used for species determination.**

The collected bark beetles (Scolytinae) were identified to species level using the key of Grüne (1979). Staphylinidae were identified to species level (apart from one individual of the sub-family Pselaphinae) by Benedikt Feldmann using the keys of (Freude et al., 1974; Lohse, 1989; Assing, 1999; 1998; Assing & Schülke 2001; 2007; 2012; Schülke & Smetana, 2015). Other beetles (including saproxylic beetles) were identified when possible to species level using the key of Lompe (2010). The bark lice (Psocoptera), snakeflies (Raphidioptera) and net-winged insects (Neuroptera) were determined to the order level and true bugs (Heteroptera) and flies (Diptera) to the family level using Stresemann (2011), Unwin (1984) and Oosterbroek (2006). Among the Hymenoptera, the chalcid wasps (Chalcidoidea) were determined to the superfamily level and braconid wasps (Braconidae), ichneumonid wasps (Ichneumonidae) and wood wasps (Xiphydriidae) to the family level using the identification key of Goulet & Huber (1993). Lepidoptera (belonging to the families Oecophoridae and Tineidae) were identified to species or genus level with the key of [www.lepiforum.de](http://www.lepiforum.de). Thysanoptera were identified to species level and assigned to ecological feeding guilds by Manfred Ulitzka using the keys of (Schliephake & Klimt, 1979; zur Strassen, 2003). Information of the ecological feeding guilds of beetles were gathered from Schmidl & Bussler (2005), Gossner et al. (2013), Horák et al. (2011), Audisio et al. (2014), Seibold et al. (2015), Wende et al. (2017), Bouget et al. (2019), and Hagge et al. (2019) and of flies from Oosterbroek (2006), Ševčík (2010), and [www.diptera.info](http://www.diptera.info).

**References for Appendix S1.**

Assing, V. F. J., Kahlen, M., Löbl, I., Lohse, G. A., Puthz, V., Schülke, M., Terlutter, H., Uhlig, M., Vogel, J., Willers, J., Wunderle, P., & Zerche, L. (1998). Familie Staphylinidae. In Lohse, G. A., & Lucht, W. H. (Eds), *Die Käfer Mitteleuropas* (Band. 15.4., pp. 119–198), Krefeld, Germany: Springer Spektrum.

Assing, V., & Schülke, M. (1999). Supplemente zur mitteleuropäischen Staphylinidenfauna (Coleoptera, Staphylinidae). *Entomologische Blätter für Biologie und Systematik der Käfer*, *95*, 1–31.

Assing, V., & Schülke, M. (2001). Supplemente zur mitteleuropäischen Staphylinidenfauna (Coleoptera, Staphylinidae). II. *Entomologische Blätter für Biologie und Systematik der Käfer*, *97*, 121–176.

Assing, V., & Schülke, M. (2007). Supplemente zur mitteleuropäischen Staphylinidenfauna (Coleoptera, Staphylinidae). III. *Entomologische Blätter für Biologie und Systematik der Käfer*, *102*, 1–78.

Assing, V., & Schülke, M. (2012). In. *Die Käfer Mitteleuropas*. Staphylinidae I. (2nd ed., Vol. 4, pp. 560), Heidelberg and Berlin: Springer Spektrum.

Audisio, P., Baviera, C., Carpaneto, G. M., Biscaccianti, A. B., Battistoni, A., Teofili, C., & Rondinini, C. (2014). Lista rossa IUCN dei coleotteri saproxilici italiani*,* (pp. 132). Rome, Italy: *Comitato Italiano IUCN e Ministero dell’Ambiente e della Tutela del Territorio e del Mare*.

Bouget, C., Brustel, H., Noblecourt, T., & Zagatti, P. (2019). *Les Coléoptères saproxyliques de France: Catalogue écologique illustré.* (pp. 744). Paris, France: Muséum national d'Histoire naturelle.

Diptera info (Paul Beuk) [www.diptera.info](http://www.diptera.info) (last accessed: Feb, 2020).

Freude, H., Harde, K. W., & Lohse, G. A. (1974). *Die Käfer Mitteleuropas* (Band. 5, pp. 1–381), Krefeld, Germany: Springer Spektrum.

Gossner, M. M., Lachat, T., Brunet, J., Isacsson, G., Bouget, C., Brustel, H., Brandl, R., Weisser, W. W., & Mueller, J. (2013). Current near to nature forest management effects on functional trait composition of saproxylic beetles in beech forests. *Conservation Biology*, *27*(3), 605–614.

Goulet, H., & Huber, J. T. (1993). Hymenoptera of the world: An identification guide to families, Ottawa, Canada: Centre for Land and Biological Resources.

Grüne, S. (1979). Handbuch zu Bestimmung der europäischen Borkenkäfer, Hannover, Germany, Verlag M. und H. Schaper.

Hagge, J., Abrego, N., Bässler, C., Bouget, C., Brin, A., Brustel, H., Christensen, M., Gossner, M. M., Heilmann-Clausen, J., Horák, J., Gruppe, A., Isacsson, G., Köhler, F., Lachat, T., Larrieu, L., Schlaghamersky, J., Thorn, S., Zapponi, L., & Müller, J. (2019). Congruent patterns of functional diversity in saproxylic beetles and fungi across European beech forests. *Journal of Biogeography*, *46*(5), 1054–1065.

Horák, J. (2011). Response of saproxylic beetles to tree species composition in a secondary urban forest area. *Urban Forestry & Urban Greening*, *10*(3), 213–222.

Lepiforum: Bestimmung von Schmetterlingen (Lepidoptera) und ihren Präimaginalstadien <http://www.lepiforum.de>

Lohse, G. A. (1989). Ergänzungen und Berichtigungen zu Band 5. 23. Familie Staphylinidae (II) (Aleocharinae). In Lohse, G. A., & Lucht, W. H. (Eds.), *Die Käfer Mitteleuropas* (1. Supplementband mit Katalogteil, pp. 185–240), Krefeld, Germany: Springer Spektrum.

Lompe, A. (2010). "Käfer Europas." http://coleo-net.de/coleo/html/impressum.htm (last accessed: January, 2020).

Oosterbroek, P. (2006). The European Families of the Diptera: Identification-Diagnosis-Biology. Leiden, The Netherlands: KNNV Publishing.

Schliephake, G., & Klimt, K. (1979). Thysanoptera, Fransenflügler. In: Senglaub, K., Hannemann, H.-J. & Schuhmann, H. (Eds), *Die Tierwelt Deutschlands und der angrenzenden Meeresteile nach ihren Merkmalen und nach ihrer Lebensweise*, (No 66, pp. 477), Jena, Germany, VEB Fischer.

Schmidl, J. V., & Bussler, H. (2004). Ökologische Gilden xylobionter Käfer Deutschlands. *Naturschutz und Landschaftsplanung*, *36*(7), 202–218.

Schülke, M., & Smetana, A. (2015). Staphylinidae,. – In: Löbl, I. & Löbl, D. (Eds.), *Catalogue of Palaearctic Coleoptera*. *Hydrophiloidea – Staphylinoidea* (Volume 2., pp. 304–1134).

Seibold, S., Brandl, R., Buse, J., Hothorn, T., Schmidl, J., Thorn, S., & Müller, J. (2015a). Association of extinction risk of saproxylic beetles with ecological degradation of forests in Europe. *Conservation Biology*, *29*(2), 382–390.

zur Strassen, R. (2003). Die terebranten Thysanopteren Europas und des Mittelmeer-Gebietes. In: Dahl, F. (Ed.) *Die Tierwelt Deutschlands*, 74. (pp. 277), Keltern, Germany: Goecke & Evers.

Stresemann, E. (2011). *Stresemann-Exkursionsfauna von Deutschland, Wirbellose: Insekten*, (11th ed., Volume 2, pp. 966), Heidelberg, Germany, Springer Spektrum.

Ševčík, J. (2010). *Czech and Slovak Diptera associated with fungi* (pp. 112). Opava, Czech Republic: Slezské zemské muzeum.

Unwin, D. M. (1984). *A key to the families of British Diptera*. Shrewsbury, UK: Field Studies Council.

Wende, B., Gossner, M. M., Grass, I., Arnstadt, T., Hofrichter, M., Floren, A., Linsenmair, K. E., Weisser, W. W., & Steffan-Dewenter, I. (2017). Trophic level, successional age and trait matching determine specialization of deadwood-based interaction networks of saproxylic beetles. *Proceedings of the Royal Society B: Biological Sciences*, *284*(1854), 20170198.

**Table S1.** Characteristics of the 25 forests examined in the city of Basel and its surroundings (northwestern Switzerland).

|  |  |  |  |  |  |  |  |  |  |  | | |
| --- | --- | --- | --- | --- | --- | --- | --- | --- | --- | --- | --- | --- |
| Forest | Coordinates | Forest history^1^ | Forest vegetation^2^ | Elevation (m a.s.l.) | Exposure^3^ | % sealed area  (r = 500 m) | Area (ha) | % Forest (r = 500 m) | Fine woody debris (m^3^/ha) | Course woody debris (m^3^/ha) | | |
|  |  |  |  |  |  |  |  |  |  | |  | |
| Bärlauchweglein | 47° 34' 7.38" N  7° 39' 4.87" E | Forest | Galio-Fagetum Pulmonarietosum | 363 | WNW | 12 | 0.8909 | 55 | 22.87 | | 7.11 | |
| Breite | 47° 33' 12.30" N  7° 36' 51.09" E | Fragment | Galio Odorati-Fagetum Cornetosum | 262 | NE | 39 | 0.7049 | 3 | 20.09 | | 13.00 | |
| Chrischona | 47° 34' 30.78” N  7° 40' 35.28” E | Forest | Galio Odorati-Fagetum Cornetosum | 487 | – | 4 | 1.4 | 79 | 8.98 | | 7.65 | |
| Erle | 47° 34' 34.58" N  7° 36' 19.23" E | Forest | Galio-Carpinetum Corydalidetosum | 269 | – | 20 | 2.1 | 27 | 6.17 | | 9.69 | |
| Friedhof-Rand | 47° 33' 50.63" N  7° 38' 42.82" E | Forest | Galio Odorati-Fagetum Pulmonarietosum | 319 | NNW | 12 | 0.4034 | 56 | 10.70 | | 8.11 | |
| Fürstensteinerstrasse | 47° 32' 12.27" N  7° 36' 5.97" E | Fragment | Galio Odorati-Fagetum Cornetosum | 321 | NE | 54 | 0.8800 | 13 | 10.85 | | 2.31 | |
| Geiser | 47° 32' 04.6" N  7° 31' 16.2" E | Forest | Galio-Fagetum Pulmonarietosum | 351 | – | 4 | 52.0 | 45 | 17.51 | | 43.43 | |
| Glögglihof | 47° 34' 53.45" N  7° 38' 51.87" E | Planted | Galio Odorati-Fagetum | 283 | – | 33 | 0.3135 | 1 | 3.25 | | 1.32 | |
| Hoelzli | 47° 31' 47.76" N  7° 35' 49.19" E | Fragment | Galio Odorati-Fagetum Typicum | 370 | E | 23 | 2.1 | 11 | 13.87 | | 9.18 | |
| Hohlweg | 47° 35' 15.09” N  7° 40' 2.90” E | Planted | Galio Odorati-Fagetum Typicum | 346 | S | 10 | 0.2800 | 21 | 5.39 | | 4.59 | |
| Jakobsbergerholz | 47° 31' 57.55" N  7° 36' 11.58" E | Fragment | Galio Odorati-Fagetum Typicum | 338 | NW | 44 | 1.4 | 19 | 7.61 | | 15.68 | |
| Klosterfiechten | 47° 31' 26.26" N  7° 35' 33.76" E | Fragment | Galio Odorati-Fagetum Typicum | 314 | – | 14 | 1.94 | 5 | 5.74 | | 3.57 | |
| Forest | Coordinates | Forest history^1^ | Forest vegetation^2^ | Elevation (m a.s.l.) | Exposure^3^ | % sealed area  (r = 500 m) | Area (m^2^) | % Forest (r = 500 m) | Fine woody debris (m^3^/ha) | Course woody debris (m^3^/ha) | | |
| Liestal | 47° 29' 10.6" N  7° 40' 43.0" E | Forest | Galio-Fagetum Pulmonarietosum | 565 | – | 0 | 359.0 | 92 | 12.99 | 6.12 | |  |
| Linsberg | 47° 34' 25.90" N  7° 39' 53.22" E | Forest | Galio Odorati-Fagetum Cornetosum | 450 | NW | 10 | 5.0 | 37 | 11.83 | 12.49 | |  |
| Maienbühl | 47° 35' 33.14” N  7° 40' 41.10” E | Forest | Galio Odorati-Fagetum Cornetosum | 473 | SW | 3 | 3.4 | 54 | 6.96 | 4.72 | |  |
| Margarethenpark | 47° 32' 30.99" N  7° 35' 2.33" E | Fragment | Galio Odorati-Fagetum Cornetosum | 299 | NNE | 37 | 1.9 | 6 | 13.91 | 4.91 | |  |
| Mooswäldli | 47° 34' 51.72" N  7° 39' 37.41" E | Fragment | Carici Remotae-Fraxinetum Typicum | 302 | – | 16 | 0.4686 | 1 | 9.29 | 25.88 | |  |
| Muttenz | 47° 30' 52.5" N  7° 38' 10.7" E | Forest | Galio-Fagetum Pulmonarietosum | 418 | – | 2 | 79.0 | 43 | 20.52 | 37.96 | |  |
| Pratteln | 47° 30' 31.4" N  7° 40' 04.3" E | Forest | Aro-Fagetum | 454 | – | 2 | 237.0 | 66 | 10.60 | 9.60 | |  |
| Salamanderweglein | 47° 35' 5.71” N  7° 40' 9.23” E | Planted | Galio Odorati-Fagetum Pulmonarietosum | 330 | SSE | 8 | 0.4061 | 34 | 10.40 | 2.97 | |  |
| Singerstrasse | 47° 32' 43.72" N  7° 36' 26.85" E | Planted | Aro-Fagetum | 276 | – | 70 | 0.1084 | 2 | 9.48 | 8.20 | |  |
| Therwil | 47° 30' 18.4" N  7° 34' 46.8" E | Forest | Galio Odorati-Fagetum Typicum | 380 | – | 2 | 41.0 | 59 | 9.68 | 18.25 | |  |
| Tramwende | 47° 34' 20.65" N  7° 37' 11.82" E | Fragment | Galio-Carpinetum Corydalidetosum | 265 | – | 12 | 0.4576 | 35 | 16.37 | 5.96 | |  |
| Wenkenköpfli | 47° 34' 29.73" N  7° 39' 29.56" E | Fragment | Galio Odorati-Fagetum Cornetosum | 384 | NW | 12 | 3.6 | 26 | 12.59 | 24.36 | |  |
| Wolfschlucht | 47° 32' 8.70" N  7° 35' 23.09" E | Fragment | Galio Odorati-Fagetum Cornetosum | 326 | E | 31 | 0.3009 | 5 | 12.10 | 1.81 | |  |

^1^ Forest = Part of a large continuous forest; Fragment = remnant of a former large continuous forest; Planted = forest site was planted after 1884

^2^Burnand, J., Hasspacher, B., 1999. Waldstandorte beider Basel. Quellen und Forschungen zur Geschichte und Landeskunde des Kanton Basel-Landschaft, Band 72. Verlag des Kantons Basel-Landschaft, Liestal.

^3^Exposure was determined for forest sites situated on a slope.

**Table S2.** Detailed results of GLM analyses examining the effects of degree of urbanisation, forest size, forest500 (percentage of forest in the surrounding 500-m radius), FWD (volume of fine woody debris), c) CWD (volume of coarse woody debris), mean decomposition stage of naturally occurring deadwood, breast height diameter (BHD), branch characteristics including total number of fungal OTUs on branches, moisture content of wood (%), wood pH, and amount of lignin on the number of individuals of total saproxylic individuals and various saproxylic arthropod groups.

|  | **Total saproxylic individuals** | **Total beetles** | **Bark beetles** | **Longhorn beetles** | **Jewel beetles** | **Total**  **flies** | **Dark-winged fungus gnats** | **Gall midges** | **Other flies** | **Moths** | **Chalcid wasps** | **Braconid wasps** | **Ichneumonid wasps** |
| --- | --- | --- | --- | --- | --- | --- | --- | --- | --- | --- | --- | --- | --- |
| Degree of urbanisation^1^ | *F*_1,23_=8.85, ***p* = 0.007** | *F*_1,23_=0.56, *p* = 0.463 | *F*_1,23_=7.20, ***p* =** **0.016** | *F*_1,23_=16.70, ***p* <0.001** | *F*_1,23_=0.02, *p* = 0.882 | *F*_1,23_=11.82, ***p* =** **0.003** | *F*_1,23_=8.53, ***p* =** **0.009** | *F*_1,23_=3.32, *p* = 0.083 | *F*_1,23_=0.71, *p* = 0.410 | *F*_1,23_=4.89, ***p* =** **0.041** | *F*_1,23_=3.65, *p* = 0.071 | *F*_1,23_=1.91, *p* = 0.182 | F_1,23_=4.60, ***p* =** **0.047** |
| Forest size^2^ | *F*_1,22_=0.53, *p* = 0.476 | *F*_1,22_=0.28, *p* = 0.601 | *F*_1,22_=0.06, *p* = 0.803 | *F*_1,22_=1.05, *p* = 0.320 | *F*_1,22_=8.07, ***p* =** **0.010** | *F*_1,22_=0.72, *p* = 0.409 | *F*_1,22_=1.02, *p* = 0.325 | *F*_1,22_=1.83, *p* = 0.191 | *F*_1,22_=1.16, *p* = 0.297 | *F*_1,22_=0.04, *p* = 0.849 | *F*_1,22_=19.66, ***p* <0.001** | *F*_1,22_=0.21, *p* = 0.648 | *F*_1,22_=2.23, *p* = 0.153 |
| Forest 500^2^ | *F*_1,21_=2.53, *p* = 0.127 | – | – | **–** | – | *F*_1,21_=4.57, ***p* =** **0.047** | *F*_1,21_=4.30, *p* = 0.052 | – | *F*_1,21_=3.07, *p* = 0.098 | *F*_1,21_=2.83, *p* = 0.111 | – | – | *F*_1,21_=3.09, *p* = 0.097 |
| FWD | – | – | *F*_1,21_=1.75, *p* = 0.203 | *F*_1,21_=12.77, ***p* =** **0.002** | – | *F*_1,20_=1.63, *p* = 0.219 | – | *F*_1,21_=4.36, *p* = 0.050 | – | *F*_1,20_=3.58, *p* = 0.076 | – | – | *F*_1,20_=4.28, ***p* =** **0.054** |
| CWD^1^ | – | – | – | *F*_1,20_=2.01, *p* = 0.173 | – | – | – | – | *F*_1,20_=1.45, *p* = 0.244 | – | *F*_1,21_=1.36, *p* = 0.259 | – | – |
|  | **Total saproxylic individuals** | **Total beetles** | **Bark beetles** | **Longhorn beetles** | **Jewel beetles** | **Total**  **flies** | **Dark-winged fungus gnats** | **Gall midges** | **Other flies** | **Moths** | **Chalcid wasps** | **Braconid wasps** | **Ichneumonid wasps** |
| Decomposition stage | – | *F*_1,21_=12.44, ***p* =** **0.002** | *F*_1,20_=1.38, *p* = 0.256 | *F*_1,19_=1.33, *p* = 0.264 | *F*_1,21_=4.64, ***p* =** **0.044** | *F*_1,19_=4.14, *p* = 0.058 | *F*_1,20_=3.28, *p* = 0.087 | *F*_1,20_=2.68, *p* = 0.117 | – | – | *F*_1,20_=18.93, ***p* =** **0.003** | *F*_1,21_=2.70, *p* = 0.116 | *F*_1,19_=2.93, *p* = 0.105 |
| Breast height diameter | – | *F*_1,20_=1.91, *p* = 0.182 | *F*_1,19_=1.40, *p* = 0.252 | – | – | – | – | – | – | – | – | – | – |
| Total number of fungal OTUs | – | – | *F*_1,18_=1.07, *p* = 0.316 | – | – | – | – | – | *F*_1,19_=1.86, *p* = 0.190 | *F*_1,19_=1.82, *p* = 0.195 | – | – | – |
| Wood moisture content (%) | *F*_1,20_=2.42, *p* = 0.136 | – | *F*_1,17_=1.51, *p* = 0.237 | – | *F*_1,20_=1.51, *p* = 0.235 | *F*_1,18_=4.88, ***p* =** **0.041** | *F*_1,19_=4.82, ***p* =** **0.042** | – | *F*_1,18_=14.54, ***p* =** **0.001** | *F*_1,17_=4.54, ***p* =** **0.048** | – | – | – |
| Wood pH | – | – | – | *F*_1,18_=4.48, ***p* = 0.049** | *F*_1,19_=3.01, *p* = 0.099 | *F*_1,17_=2.97, *p* = 0.103 | *F*_1,18_ =2.04, *p* = 0.170 | – | *F*_1,17_=1.35, *p* = 0.262 | – | *F*_1,19_=3.81, *p* = 0.066 | – | *F*_1,18_=3.82, *p* = 0.067 |
| Lignin content^1^ | – | – | – | – | – | – | – | – | – | *F*_1,16_=1.77, *p* = 0.201 | – | – | *F*_1,17_=1.76, *p* = 0.202 |

– Factor was excluded from the model due to the step-wise reduction procedure

Significant effects (*P* < 0.05) are presented in bold

^1^ = log-transformed variable

^2^ = residuals considered because of inter-correlation with degree of urbanisation

**Table S3.** Detailed results of GLM analyses examining the effects of degree of urbanisation, forest size, forest500 (percentage of forest in the surrounding 500-m radius), FWD (volume of fine woody debris), c) CWD (volume of coarse woody debris), mean decomposition stage of naturally occurring deadwood, breast height diameter (BHD), branch characteristics including total number of fungal OTUs on branches, moisture content of wood (%), wood pH, and amount of lignin on the total, beetle, flies, and moth taxonomic richness.

|  | Taxonomic richness | | |
| --- | --- | --- | --- |
| Degree of urbanisation^1^ | Beetles | Flies | Moths |
|  | *Chi^2^*_1,23_=1.90, *p* =0.168 | *Chi^2^*_1,23_=0.15, *p* =0.694 | *Chi^2^*_1,23_=0.11, *p* =0.739 |
| Forest size^2^ | *Chi^2^*_1,22_=0.50, *p* =0.480 | *Chi^2^*_1,22_=0.01, *p* =0.911 | *Chi^2^*_1,22_=0.22, *p* =0.642 |
| Forest 500^2^ | – | – | – |
| FWD | *Chi^2^*_1,21_=1.42, *p* =0.233 | – | – |
| CWD^1^ | – | – | – |
| Decomposition stage | *Chi^2^*_1,20_=1.64, *p* =0.201 | – | – |
| Breast height diameter | – | – | – |
| Total number of fungal OTUs | – | – | *Chi^2^*_1,23_=1.16, *p* =0.281 |
| Wood moisture content (%) | – | *Chi^2^*_1,21_=1.06, *p* =0.302 | – |
| Wood pH | – | – | – |
| Lignin content^1^ | – | – | – |

– Factor was excluded from the model due to the step-wise reduction procedure

Significant effects (*P* < 0.05) are presented in bold

^1^ = log-transformed variable

^2^ = residuals considered because of inter-correlation with degree of urbanisation

**Table S4.** Table depicting all identified saproxylic individuals assigned to their ecological feeding guilds. The saproxylic individuals were classified into 5 feeding guilds. 1) mycetophagous (consuming fungi or mouldy material), 2) saprophagous (feeding on decaying organic material), 3) xylophagous (primary consumers of woody material), 4) predatory (predators on other saproxylics), and 5) parasitic (developing within other saproxylic larvae or parasitoid larvae feed externally on saproxylics).

The ecological feeding guilds information for the beetles were derived from Schmidl & Bussler (2005), Horak et al. (2011); Gossner et al. (2013); Audisio et al. (2014); Seibold et al. (2015); Wende et al. (2017); Bouget et al. (2019); Hagge et al. (2019). Beetles that were not present in the German list (Schmidl & Bussler, 2005) or German databases/publications but were present in the Italian (Audisio et al., 2014) or French (Bouget et al., 2019) saproxylic red lists, or in Horak et al. (2011) were included as saproxylic beetles. The ecological feeding guilds for Thysanoptera were obtained from Schliephake & Klimt (1979) and zur Strassen (2003), those for flies from Oosterbroek (2006), Sevcick (2010) and [www.diptera.info](http://www.diptera.info). The Hymenoptera were assigned to feeding guilds following Goulet & Huber (1993). The other insects were assigned to feeding guilds following Stresemann (2011). The articles cited in this table are listed in the reference list of Appendix S1.

| **Insect group** | **Species/Genus/Sub-family/Family/Superfamily** | **Ecological feeding guild** | **Number of individuals** |
| --- | --- | --- | --- |
| Curculionidae | *Acalles micros* | xylophagous | 2 |
| Curculionidae | *Kyklioacalles roboris* | xylophagous | 4 |
| Curculionidae | *Trachodes hispidus* | xylophagous | 17 |
| Curculionidae | *Magdalis flavicornis* | xylophagous | 1 |
| Curculionidae | *Rhyncolus ater* | xylophagous | 2 |
| Curculionidae | *Euophryum confine* | xylophagous | 1 |
| Curculionidae Scolytinae | *Taphrorychus bicolor* | xylophagous | 14401 |
| Curculionidae Scolytinae | *Ernoporicus fagi* | xylophagous | 657 |
| Curculionidae Scolytinae | *Scolytus intricatus* | xylophagous | 2508 |
| Curculionidae Scolytinae | *Xylosandrus germanus* | mycetophagous | 31 |
| Curculionidae Scolytinae | *Xyleborus monographus* | mycetophagous | 58 |
| Salpingidae | *Salpingus ruficollis* | predatory | 2 |
| Salpingidae | *Salpingus planirostris* | predatory | 61 |
| Salpingidae | *Vincenzellus ruficollis* | predatory | 4 |
| Cleridae | *Opilo molis* | predatory | 2 |
| Elateridae | *Ampedus sanguinolentus* | xylophagous | 1 |
| Laemophloeidae | *Laemophloeus monilis* | Predatory larvae | 1 |
| Laemophloeidae | *Placonotus testaceus* | Predatory larvae | 3 |
| Laemophloeidae | *Cryptolestes duplicatus* | Predatory larvae | 11 |
| **Insect group** | **Species/Genus/Sub-family/Family/Superfamily** | **Ecological feeding guild** | **Number of individuals** |
| Silvanidae | *Uleiota planata* | predatory | 26 |
| Silvanidae | *Silvanus unidentatus* | predatory | 51 |
| Silvanidae | *Silvanus bidentatus* | predatory | 41 |
| Monotomidae | *Rhizophagus bipustulatus* | predatory | 9 |
| Monotomidae | *Rhizophagus perforatus* | predatory | 1 |
| Monotomidae | *Monotoma longicollis* | mycetophagous | 1 |
| Trogositidae | *Nemozoma elongatum* | predatory | 22 |
| Anthribidae | *Platystomos albinus* | xylophagous | 2 |
| Anthribidae | *Pseudeuparius sepicola* | xylophagous | 1 |
| Melyridae | *Dasytes plumbeus* | predatory | 33 |
| Melyridae | *Dasytes aeratus* | predatory | 1 |
| Melyridae | *Daystes caeruleseus* | predatory | 11 |
| Scraptiidae | *Anaspis flava* | xylophagous | 19 |
| Scraptiidae | *Anaspis frontalis* | xylophagous | 1 |
| Anobiidae | *Anobium punctatum* | xylophagous | 1 |
| Anobiidae | *Hemicoelus fulvicornis* | xylophagous | 1 |
| Anobiidae | *Xestobium plumbeum* | xylophagous | 1 |
| Anobiidae | *Ernobius angusticollis* | xylophagous | 1 |
| Mycetophagidae | *Litargus connexus* | mycetophagous | 430 |
| Mycetophagidae | *Mycetophagus atomarius* | mycetophagous | 2 |
| Mycetophagidae | *Typhaea stercorea* | mycetophagous | 2 |
| Mycetophagidae | *Berginus tamarisci* | mycetophagous | 3 |
| Zoopheridae | *Synchita undata* | mycetophagous | 1 |
| Malachiidae | *Sphinginus lobatus* | Predatory larvae | 1 |
| Throscidae | *Aulonothroscus brevicollis* | saprophagous scavenger | 17 |
| Nitidulidae | *Epuraea rufomarginata* | mycetophagous | 2 |
| Leiodidae | *Anisotoma* sp. | mycetophagous | 1 |
| Corylophidae | *Arthrolips* sp. | mycetophagous | 1 |
| Corylophidae | *Sericoderus* *lateralis* | saprophagous scavenger | 4534 |
| Latridiidae | *Corticaria* sp. | mycetophagous | 8 |
| Latridiidae | *Stephostethus alternans* | mycetophagous | 3 |
| Latridiidae | *Dienerella clathrata* | mycetophagous | 10 |
| Latridiidae | *Cartodere nodifer* | mycetophagous | 221 |
| **Insect group** | **Species/Genus/Sub-family/Family/Superfamily** | **Ecological feeding guild** | **Number of individuals** |
| Latridiidae | *Latridius* sp. | mycetophagous | 93 |
| Cryptophagidae | *Atomaria* sp. | mycetophagous | 255 |
| Cryptophagidae | *Cryptophagus* sp. | mycetophagous | 11386 |
| Cerambycidae | *Pogonocherus hispidulus* | xylophagous | 230 |
| Cerambycidae | *Leiopus nebulosus* aggr. | xylophagous | 392 |
| Cerambycidae | *Mesosa nebulosa* | xylophagous | 6 |
| Cerambycidae | *Clytus arietis* | xylophagous | 1 |
| Cerambycidae | *Xylotrechus antilope* | xylophagous | 1 |
| Cerambycidae | *Phymatodes testaceus* | xylophagous | 126 |
| Cerambycidae | *Grammoptera ruficornis* | xylophagous | 4 |
| Cerambycidae | *Anaesthetis testacea* | xylophagous | 1 |
| Buprestidae | *Agrilus angustulus* | xylophagous | 522 |
| Buprestidae | *Agrilus sulcicollis* | xylophagous | 11 |
| Buprestidae | *Agrilus olivicolor* | xylophagous | 926 |
| Buprestidae | *Agrilus graminis* | xylophagous | 2 |
| Staphylinidae | *Phloeocharis subtilissima* | predatory | 10 |
| Staphylinidae | *Phloeonomus punctipennis* | predatory | 16 |
| Staphylinidae | *Carpelimus gracilis* | saprophagous | 1 |
| Staphylinidae | *Tachyporus obtusus* | predatory | 1 |
| Staphylinidae | *Oligota granaria* | predatory | 25 |
| Staphylinidae | *Oligota parva* | predatory | 3 |
| Staphylinidae | *Cypha apicalis* | predatory | 1 |
| Staphylinidae | *Anomognathus cuspidatus* | predatory | 11 |
| Staphylinidae | *Leptusa fumida* | predatory | 7 |
| Staphylinidae | *Leptusa ruficollis* | predatory | 22 |
| Staphylinidae | *Atheta coriaria* | predatory | 304 |
| Staphylinidae | Pselaphinae sp. | predatory | 1 |
| Carabidae | *Dromius quadrimaculatus* | predatory | 7 |
|  |  |  |  |
| **Parasitoid wasps** |  |  |  |
| Chalcidodea | Chalcid wasps | parasitoid | 5148 |
| Braconidae | Braconid wasps | parasitoid | 5675 |
| Ichneumonidae | Ichneumonid wasps | parasitoid | 431 |
| **Insect group** | **Species/Genus/Sub-family/Family/Superfamily** | **Ecological feeding guild** | **Number of individuals** |
| **Moths** |  |  |  |
| Oecophoridae | *Oecophora bractella* | mycetophagous | 30 |
| Oecophoridae | *Metalampra italica* | mycetophagous | 1299 |
| Oecophoridae | *Shiffermuelleria schaefferella* | mycetophagous | 2 |
| Oecophoridae | *Epicallima formosella* | mycetophagous | 23 |
| Tineidae | *Nemapogon* spp. | mycetophagous | 201 |
|  |  |  |  |
| **Diptera** |  |  |  |
| Cecidomyiidae | Sub-family Lestremiinae and Micromyiinae (wood midges) | mycetophagous | 44474 |
| Sciaridae | *Trichosia sp.* | mycetophagous | 88230 |
| Mycetophilidae |  | mycetophagous | 466 |
| Phoridae | *Megaselia* sp. | unknown | 181 |
| Drosophilidae | *Stegana* sp. | saprophagous | 91 |
| Chloropidae | *Gaurax fascipes* | mycetophagous | 187 |
| Scatopsidae | *Coboldia fuscipes* | saprophagous | 2787 |
| Syrphidae | *Cheilosia longula* | mycetophagous | 1 |
| Tipulidae |  | phyto saprophagous | 1 |
| Anthomyiidae |  | saprophage | 28 |
| Stratiomyidae | *Pachygaster atra* | predatory necrophagous | 1 |
| Dolichopodidae | *Medetera* sp. | predatory | 56 |
| Lauxaniidae | *Homoneura interstincta* | Saprophagous | 2 |
|  |  |  |  |
| **Saproxylic Heteroptera** |  |  |  |
| Anthocoridae | *Xylocoris cursitans* | predatory | 215 |
| Aradidae | *Aneurus laevis* | mycetophagous | 1 |
|  |  |  |  |
| **Other saproxylics** |  |  |  |
| Psocoptera | *Caecilius fuscopterus* and *Trichadenotecnum* sp. | phyto saprophagous | 6324 |
| Thysanoptera | *Xylaplothrips fulginosus* | mycetophagous | 4 |
| Thysanoptera | *Hoplothrips corticis* | mycetophagous | 23 |
| Thysanoptera (Phlaeothripidae) | *Poecilothrips albopictus* | mycetophagous | 44 |
| **Insect group** | **Species/Genus/Sub-family/Family/Superfamily** | **Ecological feeding guild** | **Number of individuals** |
| Thysanoptera | *Aeolothrips versicolor* | predatory | 1 |
| Raphidioptera |  | predatory | 1 |
| Neuroptera | larvae | predatory | 2 |
| Xiphydridae |  | xylophagous | 14 |
| **Total number of saproxylic insect individuals** | |  | **193534** |

**Table S5.** Results of GLM analyses examining the effects of degree of urbanisation, forest size, forest500 (percentage of forest within a radius of 500 m), FWD (volume of fine woody debris), CWD (volume of coarse woody debris), mean decomposition stage of naturally occurring deadwood, breast height diameter (BHD), branch characteristics including total number of fungal OTUs on branches, moisture content of wood (%), wood pH, and amount of lignin on the proportion of insect individuals belonging to different feeding guilds.

|  | Proportion of mycetophagous individuals | Proportion of saprophagous individuals | Proportion of all xylophagous individuals | Proportion of all predatory individuals | Proportion of parasitic wasps |
| --- | --- | --- | --- | --- | --- |
| Degree of urbanisation^1^ | *F*_1,23_=3.29, *p* = 0.088 | *F*_1,23_=24.48, ***p* <** **0.001** | *F*_1,23_=0.29, *p* = 0.596 | *F*_1,23_=0.002, *p* = 0.962 | *F*_1,23_=0.24, *p* = 0.630 |
| Forest size^2^ | *F*_1,22_=1.18, *p* = 0.293 | *F*_1,22_=18.47, ***p* <** **0.001** | *F*_1,22_=0.0002, *p* = 0.990 | *F*_1,22_=0.72, *p* = 0.407 | *F*_1,22_=0.41, *p* = 0.532 |
| Forest 500^2^ | *F*_1,21_=6.12, ***p* = 0.025** | *F*_1,21_=8.25, ***p* = 0.011** | *F*_1,21_=1.01, *p* = 0.328 | – | *F*_1,21_=1.10, *p* = 0.306 |
| FWD | *F*_1,20_=3.13, *p* = 0.096 | – | *F*_1,20_=1.35, *p* = 0.262 | *F*_1,21_=1.29, *p* = 0.271 | – |
| CWD^1^ | *F*_1,19_=1.63, *p* = 0.220 | *F*_1,20_=4.18, *p* = 0.057 | – | – | – |
| Decomposition stage | *F*_1,18_=16.99, ***p* < 0.001** | *F*_1,19_=10.81, ***p* =** **0.004** | *F*_1,19_=3.05, *p* = 0.099 | *F*_1,20_=2.00, *p* = 0.174 | *F*_1,20_=6.36, ***p* =** **0.020** |
| Breast height diameter | – | – | – | – | – |
| Total number of fungal OTUs | – | – | – | – | – |
| Wood moisture content (%) | *F*_1,17_=12.52, ***p* = 0.003** | *F*_1,17_=9.35, ***p* = 0.007** | *F*_1,18_=3.88, *p* = 0.065 | *F*_1,19_=2.63, *p* = 0.122 | – |
| Wood pH | *F*_1,16_=2.43, *p* = 0.139 | – | *F*_1,17_=2.43, *p* = 0.137 | *F*_1,18_=2.57, *p* = 0.127 | – |
| Lignin content^1^ | – | *F*_1,17_=1.19, ***p* = 0.290** | – | – | – |

– Factor was excluded from the model due to the step-wise reduction procedure

Significant effects (*P* < 0.05) are presented in bold

^1^ = log-transformed variable

^2^ = residuals considered because of inter-correlation with degree of urbanisation

**Table S6.** Detailed results of GLM analyses examining the effects of degree of urbanisation, forest size, forest500 (percentage of forest in the surrounding 500-m radius), FWD (volume of fine woody debris), c) CWD (volume of coarse woody debris), mean decomposition stage of naturally occurring deadwood, breast height diameter (BHD), branch characteristics including total number of fungal OTUs on branches, moisture content of wood (%), wood pH, and amount of lignin on the proportion of total and beetle mycetophagous, saprophagous, xylophagous, and predatory taxonomic richness.

|  | Proportion of taxonomic richness | | | | | | | |
| --- | --- | --- | --- | --- | --- | --- | --- | --- |
| Degree of urbanisation^1^ | Total mycetophagous | Mycetophagous beetles | Total saprophagous | Saprophagous beetle | Total xylophagous | Xylophagous beetles | Total predatory | Predatory beetles |
|  | *Chi^2^*_1,23_=0.16, *p* =0.687 | *Chi^2^*_1,23_=1.38, *p* =0.240 | *Chi^2^*_1,23_=0.86, *p* =0.354 | *Chi^2^*_1,23_=0.56, *p* =0.455 | *Chi^2^*_1,23_=1.02, *p* =0.312 | *Chi^2^*_1,23_=0.97, *p* =0.324 | *Chi^2^*_1,23_=0.17, *p* =0.682 | *Chi^2^*_1,23_=0.25, *p* =0.617 |
| Forest size^2^ | *Chi^2^*_1,22_=0.10, *p* =0.755 | *Chi^2^*_1,22_=0.80, *p* =0.372 | *Chi^2^*_1,22_=0.73, *p* =0.394 | *Chi^2^*_1,22_=0.002, *p* =0.963 | *Chi^2^*_1,22_=0.02, *p* =0.891 | *Chi^2^*_1,22_=0.06, *p* =0.806 | *Chi^2^*_1,22_=1.45, *p* =0.229 | *Chi^2^*_1,22_=1.10, *p* =0.294 |
| Forest 500^2^ | – | – | – | – | – | – | – | *Chi^2^*_1,21_=1.07, *p* =0.302 |
| FWD | – | – | – | – | – | – | – | – |
| CWD^1^ | – | – | – | – | – | – | – | – |
| Decomposition stage | – | – | – | – | – | – | – | – |
| Breast height diameter | – | – | – | *Chi^2^*_1,21_=1.28, *p* =0.257 | – | – | – | – |
| Total number of fungal OTUs | – | – | *Chi^2^*_1,21_=1.19, *p* =0.276 | – | – | – | – | – |
| Wood moisture content (%) | – | – | – | – | – | – | – | – |
| Wood pH | – | – | – | – | – | – | – | – |
| Lignin content^1^ | – | – | – | – | – | – | – | – |

- Factor was excluded from the model due to the step-wise reduction procedure, Significant effects (*P* < 0.05) are presented in bold,

^1^ = log-transformed variable, ^2^ = residuals considered because of inter-correlation with degree of urbanisation

**Table S7.** Summary of the GLM analyses examining the effects of degree of urbanisation, forest size, forest500 (percentage of forest in the surrounding 500-m radius), FWD (volume of fine woody debris), c) CWD (volume of coarse woody debris), decomposition stage of naturally occurring deadwood, breast height diameter (BHD), branch characteristics including moisture content of wood (%), wood pH and lignin content on the number of total fungal species (OTUs) on the branches exposed and separately the number of fungal species on both the beech and oak branches exposed in the 25 forests.

|  | Total number of fungal species (OTUs) on the branch bundles^1^ | Number of fungal species (OTUs) on beech branches^1^ | Number of fungal species (OTUs) on oak branches^1^ |
| --- | --- | --- | --- |
| Degree of urbanisation | *Chi^2^*_1,23_=6.31, ***p* =** **0.012** | *Chi^2^*_1,23_=0.90, *p* = 0.342 | *Chi^2^*_1,23_=8.84, ***p* =** **0.003** |
| Forest size | *Chi^2^*_1,22_=0.98, *p* = 0.323 | *Chi^2^*_1,22_=1.61, *p* = 0.204 | *Chi^2^*_1,22_=1.12, *p* = 0.290 |
| Forest 500 | *Chi^2^*_1,21_=2.00, *p* = 0.157 | – | *Chi^2^*_1,21_=3.01, *p* = 0.083 |
| FWD | *Chi^2^*_1,20_=4.21, ***p* =** **0.040** | *Chi^2^*_1,21_=2.14, *p* = 0.144 | *Chi^2^*_1,20_=4.91, ***p* =** **0.027** |
| CWD | – | – | – |
| Decomposition stage | – | – | – |
| Breast height diameter | – | – | – |
| Wood moisture content (%) | – | *Chi^2^*_1,20_=1.45, *p* = 0.229 | – |
| Wood pH | – | – | – |
| Lignin content | – | *Chi^2^*_1,19_=1.31, *p* = 0.253 | – |

^1^ = GLM model with poisson distributed errors

^2^ = GLM model with quasipoisson distributed errors

– Factor was excluded from the model due to the step-wise reduction procedure

Significant effects (*P* < 0.05) are presented in bold


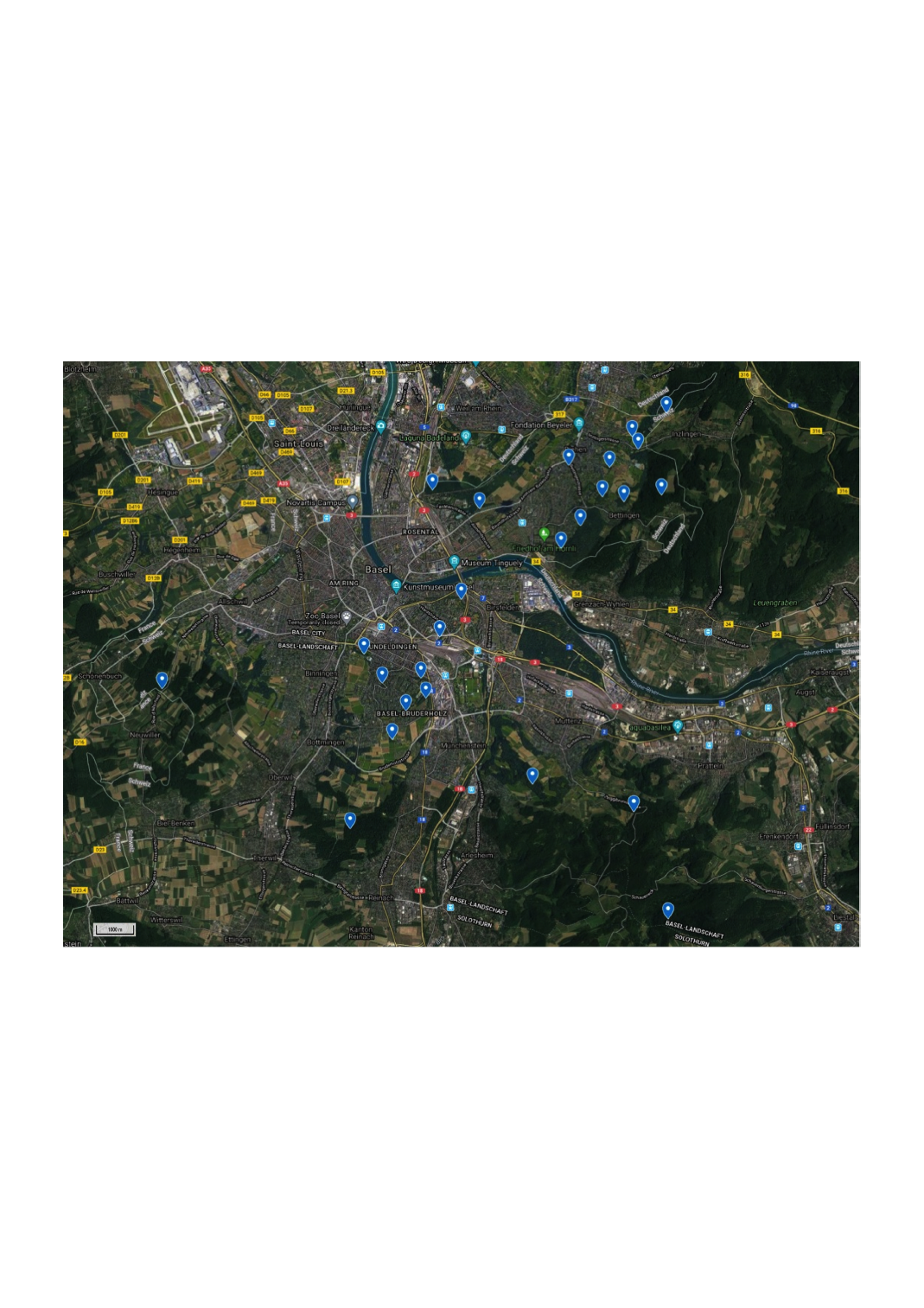


**Figure S1** The 25 forest sites (marked in dark blue) that were investigated in Basel and surroundings situated in varying degrees of

urbanisation and consisting of a range of forest sizes (Source: Google Earth, 07.2018).


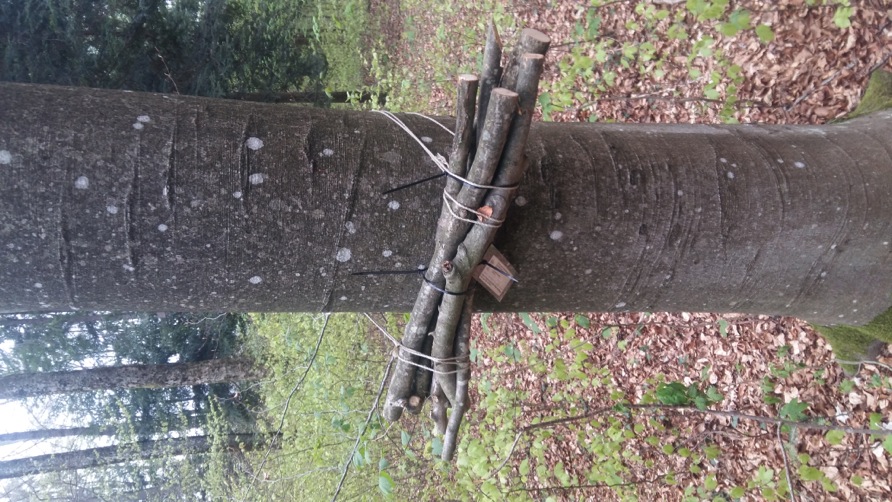


**Figure S2** Branch bundle attached to a beech stem at a height of 1.5 m.


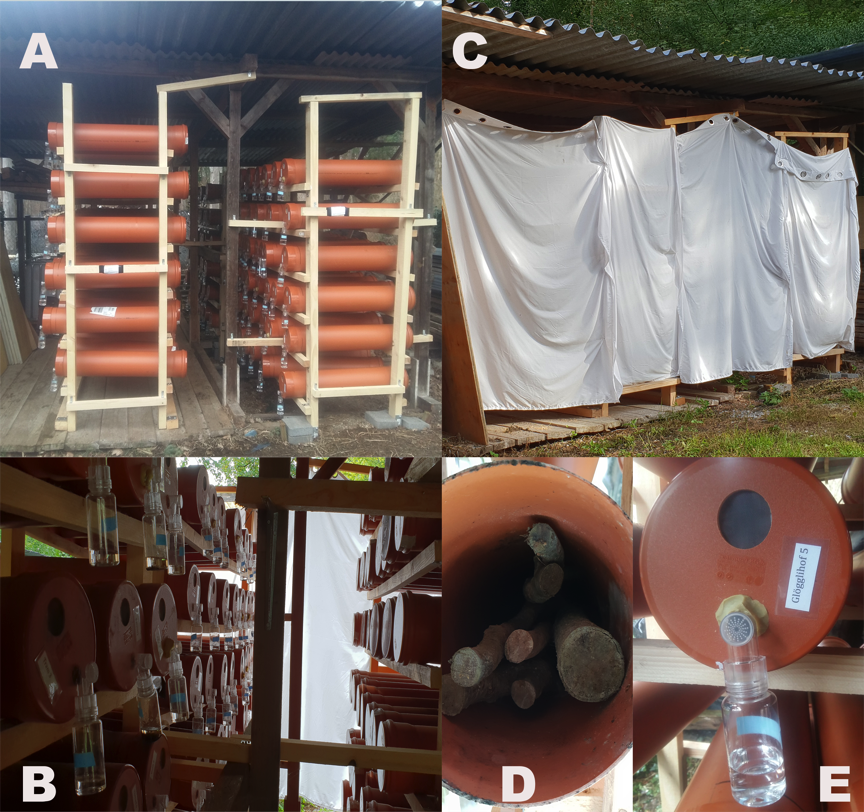


**Figure S3. Emergence traps.** A), B) emergence traps wooden frame. C) white cloth in front of the emergence traps to block off the direct midday sunlight, D) branches within an emergence trap, and E) the lid set up of the emergence trap with a 51 mm hole covered with 0.5 mm mesh size for ventilation and the collection bottle (FJ Technology Co., Ltd.) with 70% ethanol, which was emptied and refilled every month.
